# Coronary artery disease genes *SMAD3* and *TCF21* promote opposing interactive genetic programs that regulate smooth muscle cell differentiation and disease risk

**DOI:** 10.1371/journal.pgen.1007681

**Published:** 2018-10-11

**Authors:** Dharini Iyer, Quanyi Zhao, Robert Wirka, Ameay Naravane, Trieu Nguyen, Boxiang Liu, Manabu Nagao, Paul Cheng, Clint L. Miller, Juyong Brian Kim, Milos Pjanic, Thomas Quertermous

**Affiliations:** 1 Department of Medicine and Cardiovascular Institute, Stanford University School of Medicine, Stanford, CA, United States of America; 2 Departments of Public Health Sciences, Biochemistry and Genetics, and Biomedical Engineering, University of Virginia, Charlottesville, VA, United States of America; University of Pennsylvania, UNITED STATES

## Abstract

Although numerous genetic loci have been associated with coronary artery disease (CAD) with genome wide association studies, efforts are needed to identify the causal genes in these loci and link them into fundamental signaling pathways. Recent studies have investigated the disease mechanism of CAD associated gene *SMAD3*, a central transcription factor (TF) in the TGFβ pathway, investigating its role in smooth muscle biology. In vitro studies in human coronary artery smooth muscle cells (HCASMC) revealed that SMAD3 modulates cellular phenotype, promoting expression of differentiation marker genes while inhibiting proliferation. RNA sequencing and chromatin immunoprecipitation sequencing studies in HCASMC identified downstream genes that reside in pathways which mediate vascular development and atherosclerosis processes in this cell type. HCASMC phenotype, and gene expression patterns promoted by SMAD3 were noted to have opposing direction of effect compared to another CAD associated TF, TCF21. At sites of SMAD3 and TCF21 colocalization on DNA, SMAD3 binding was inversely correlated with TCF21 binding, due in part to TCF21 locally blocking chromatin accessibility at the SMAD3 binding site. Further, TCF21 was able to directly inhibit SMAD3 activation of gene expression in transfection reporter gene studies. In contrast to *TCF21* which is protective toward CAD, *SMAD3* expression in HCASMC was shown to be directly correlated with disease risk. We propose that the pro-differentiation action of *SMAD3* inhibits dedifferentiation that is required for HCASMC to expand and stabilize disease plaque as they respond to vascular stresses, counteracting the protective dedifferentiating activity of *TCF21* and promoting disease risk.

## Author summary

Coronary artery disease (CAD) is the worldwide leading cause of death. The majority of risk for CAD is genetic in nature, i.e., a feature of the genetic information that is transmitted to each individual from both parents, and primarily affects the disease processes in the blood vessel wall that regulate the disease molecular pathways. Modern genetic approaches have allowed mapping of the regions of the human genome that encode information that mediates this risk. The *SMAD3* gene has been identified through these studies, a known master regulatory of other genes and molecular pathways, and we have investigated the functions of this gene that are important for disease risk. *SMAD3* affects basic functions of a cellular component of the vessel wall, the smooth muscle cell (SMC), that is responsible for responding to vascular stresses to heal the lesions that are produced in conjunction with elevated lipids and other classic risk factors. Studies reported here show that *SMAD3* actually inhibits the cellular processes that allow SMC to repair the vascular lesions, and its expression is promoted by the disease related variable sequences in the disease associated regions of the genome. *SMAD3* is opposed by another CAD gene, *TCF21*, that functions to block the effects of *SMAD3* expression, and these studies identify genetic mechanisms by which this is done. Thus, these studies identify an interactive pathway that directly contributes to disease risk, and the ability to block *SMAD3* or promote *TCF21* function could be exploited to inhibit vascular events such as myocardial infarction.

## Introduction

Coronary artery disease (CAD) is the worldwide leading cause of death [1]. Numerous genetic loci have been associated with CAD with genome-wide association studies (GWAS) [2–8] and point to common inherited variation that mediates the genetic risk for this disease. Unfortunately, a majority of the identified causal variation resides outside of protein coding exons, in regulatory regions of the genome that are poorly understood [9], and further efforts are required to understand the mechanisms of disease association. Thus, ongoing efforts are required to identify causal genes in these loci and link them to fundamental signaling pathways that may be targeted for therapeutic benefit.

One molecular pathway that appears to be highly represented among the causal genes identified in CAD loci is constituted with members of the TGFβ superfamily [10]. Also, our unbiased genome-wide studies of chromosomal accessibility and epigenome mapping in human coronary artery smooth muscle cells (HCASMC) have identified a significant enrichment for CAD loci in chromosomal regions affected by TGFβ signaling in this cell type [11]. TGFβ signaling controls a diverse set of cellular processes, including proliferation, cell-cell recognition, differentiation, and specification of developmental fate, during embryogenesis as well as in mature tissues [12–18]. One TGFβ family member, SMAD3, has been experimentally linked to atherosclerosis [19, 20], recently associated with CAD, and implicated with genomic and functional studies as the causal gene at 15q22.33 by this and another laboratory [11, 21]. These studies implicate this critical component of the canonical TGFβ pathway in CAD, although the disease relevant cell type and mechanism of effect remain unclear.

There are considerable data that link SMAD3 to developmental and disease processes in the smooth muscle cell (SMC) component of the coronary circulation. In embryonic development, TGFβ signaling plays a key role in SMC differentiation from the mesoderm and the intimately related process of vascular wall development [22–25]. Importantly, TGFβ mediates epithelial mesenchymal transition (EMT) in development of the coronary circulation, promoting epicardial cell migration into the myocardium and formation of coronary artery smooth muscle cells [26–28]. Data also suggests that SMAD3 regulates fundamental SMC processes that are relevant to vascular disease risk. *SMAD3* mutations have been linked to the syndromic disease Aneurysms Osteoarthritis Syndrome that is characterized by large vessel aneurysms that primarily result from loss of SMC and related structural matrix components [29]. Relevant to atherosclerosis and CAD, SMAD3 has been shown to directly bind the SMC lineage determining transcription factor myocardin (*MYOCD*) to regulate transcription of differentiation factors [30] and linked to differentiative and anti-proliferative effects in a number of smooth muscle cell models [23–25, 31]. Interestingly, these actions appear to directly oppose those of another CAD associated gene *TCF21*. *TCF21* promotes dedifferentiation, proliferation and migration of HCASMC, broadly promoting phenotypic switching, an epigenetic process that is hypothesized to reduce disease risk [32–34]. Taken together, these findings suggest that cell state changes in HCASMC are a critical aspect of CAD pathophysiology, that dedifferentiation is a critical protective function against vascular destabilization, and predict that SMAD3 and TCF21 have opposing functional roles in regulating the phenotype of HCASMC.

In studies reported here, we have focused on SMAD3 as a key factor in the pathophysiology of CAD, because of its localization in a CAD associated locus [4, 35], its central role as an effector in the TGFβ pathway [36–40], and its function as a transcriptional regulator [30, 39, 41, 42]. In vitro functional studies along with RNAseq and ChIPseq analyses establish that SMAD3 elicits a pro-differentiation phenotype in HCASMC, opposing the functions of the CAD associated factor TCF21 to promote CAD risk [32–34].

## Results

### SMAD3 promotes differentiation and migration but inhibits proliferation of HCASMC

**Fig 1:**
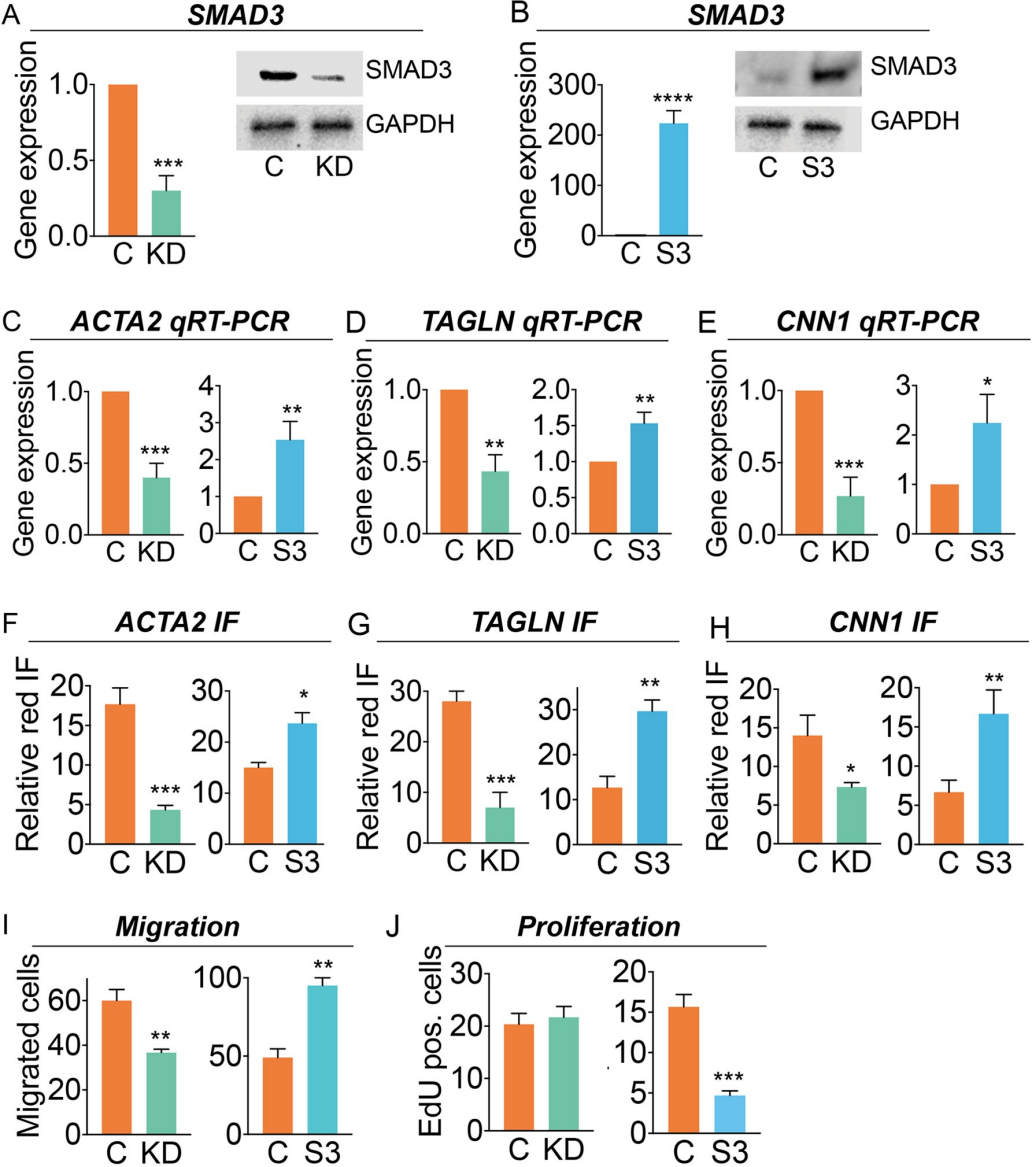
SMAD3 promotes expression of HCASMC differentiation markers. A, B) HCASMC were treated with *SMAD3* specific (KD, green bars) or scrambled sequence (C) siRNA molecules, or transfected with a *SMAD3* encoding expression plasmid (S3, blue bars) or control plasmid (C). *SMAD3* expression was evaluated by qRT-PCR and western blot analysis with GAPDH protein levels evaluated as a control. C, D, E) Gene expression was quantified for HCASMC lineage markers *ACTA2*, *TAGLN and CNN1* by qRT-PCR in cells with SMAD3 knockdown and over-expression, shown here, and western blot (S1A Fig). F, G, H) Differentiation marker expression was also evaluated by quantitative immunofluorescence (IF) for cell-specific genes *ACTA2*, *TAGLN* and *CNN1* by SMAD3 knockdown or increased expression. I) HCASMC with SMAD3 knockdown or over-expression were evaluated for HCASMC migratory activity with a gap closure assay. J) To evaluate the effect of *SMAD3* expression on cell division, we labeled cells with EdU (5-ethynyl-2’ -deoxyuridine), imaged for nuclear fluorescence, and quantified the relative number of EdU positive DAPI stained cells for HCASMC undergoing SMAD3 knockdown or increased expression. *p*-values: ****, *p*<0.0001; ***, *p*<0.001; **, *p*<0.01; *, *p*<0.05.

Given the role of TGFβ in differentiation of epicardial precursor cells to the coronary smooth muscle cell lineage during embryogenesis [26–28], we specifically investigated the expression of HCASMC marker genes in *SMAD3* siRNA knockdown experiments. Comparison of HCASMC transfected with a specific *SMAD3* siRNA or an RNA with scrambled sequence showed that a significant decrease in *SMAD3* mRNA levels (1.0 vs. 0.30, *p*<0.001) and a 65% decrease in protein expression by quantitative western analysis (Fig 1A). This was associated with decreased mRNA levels for SMC markers *ACTA2* (1.0 vs. 0.4, *p*<0.001,) *TAGLN* (1.0 vs. 0.43, *p*<0.01), and *CNN1* (1.0 vs 0.27, p<0.001) as well as decreased protein levels for ACTA2 and TAGLN (Fig 1C, 1D and 1E, S1A Fig). Similar experiments were performed with over-expression of *SMAD3* in HCASMC, achieved by transfection of these cells with a *SMAD3* expression construct, as assessed by mRNA levels (1.0 vs. 223.3, *p*<0.0001) with an average 11-fold increase in protein expression as determine by quantitative western analysis (Fig 1B). Confirming an opposite effect on *ACTA2*, *TAGLN*, *and CNN1* expression, increases were observed in mRNA levels for these two genes (1.0 vs. 2.53, *p*<0.01, 1.0 vs. 1.50, *p*<0.01, and 1.0 vs. 2.24, *p*<0.05 respectively), as well as increased protein levels (Fig 1C, 1D and 1E, S1A Fig). In a separate type of assay, fluorescence was quantified for HCASMC expressing either increased or decreased SMAD3. These studies also indicated that decreased levels of SMAD3 were associated with decreased expression of *ACTA2* (17.7, vs. 4.3, p,0.001), *TAGLN* (28 vs. 7, *p*<0.001), and *CNN1* (14 vs. 7.3, *p*<0.05) while increased SMAD3 promoted increased expression of *ACTA2* (15.0 vs 23.7, *p*<0.01), *TAGLN* (12.7 vs 29.7, *p*<0.01) and *CNN1* (6.67 vs, 16.7, *p*<0.01) marker genes (Fig 1F, 1G and 1H, S1B Fig and S1C Fig).

The effect of SMAD3 on cellular migration and proliferation of HCASMC was also investigated. Employing a wound closure assay of migration, *SMAD3* siRNA knockdown significantly decreased the surface area covered by the cells after 24 hours of incubation (60 vs. 36.67 for control cells, *p*<0.01) (Fig 1I, S1D Fig). Over-expression of *SMAD3* produced a significant increase in migratory activity (49 vs. 95.8 control cells, *p*<0.01). Although seemingly inconsistent with the differentiation effect in HCASMC, SMAD3 has been previously shown to promote migration in culture model systems [43]. The ability of SMAD3 to regulate cell cycle in the cultured HCASMC was evaluated with a high sensitivity EdU assay. Knockdown of *SMAD3* did not show an effect while over-expression produced a significant decrease in cellular proliferation (15.67 vs. 4.67 control cells, *p*<0.001) (Fig 1J, S1E Fig).

### RNAseq studies identify developmental and disease pathways in HCASMC downstream of SMAD3

**Fig 2:**
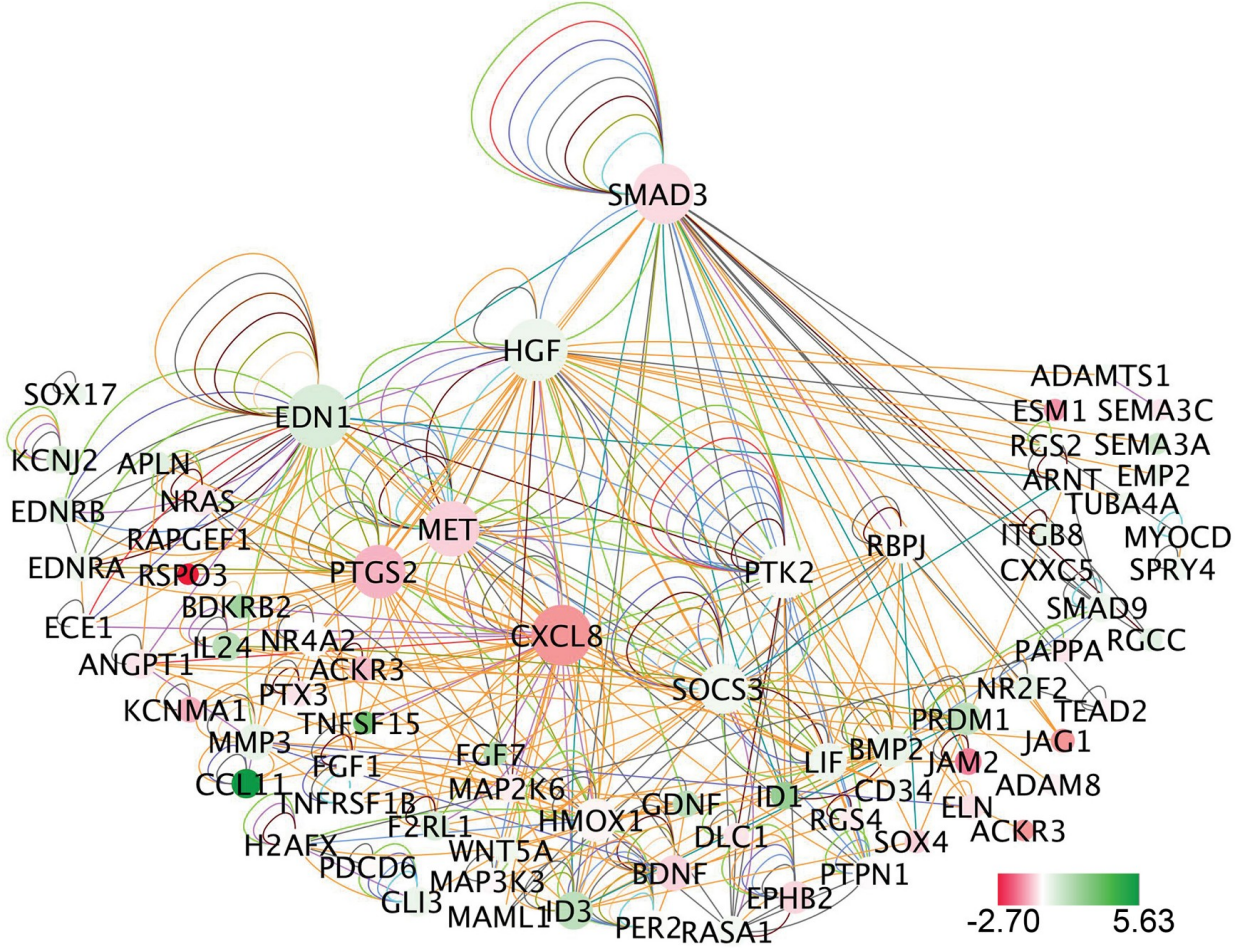
*SMAD3* “vascular development” network built with differential gene expression data from si*SMAD3* RNAseq studies in HCASMC. Interaction of the network nodes identified through enrichment of differentially expressed genes in functionally annotated categories was visualized with Cytoscape. Node color was mapped to log2 fold-change with red representing genes that are downregulated along with *SMAD3* and green representing genes that are upregulated, and node size mapped to the number of interactions with other genes. Edges are colored to distinguish types of interactions: bright green edges represent activation, neon carrot—expression, red—inhibition, purple—localization, blue—molecular cleavage, malibu light blue—phosphorylation, persian green- protein-DNA interactions, dove grey—protein-protein interactions, saddle brown—reaction, lonestar red—regulation of binding, dusty gray—transcription, peach orange—translocation, aqua blue—ubiquitination.

To gain insights into the role of *SMAD3* expression in HCASMC, and support the in vitro functional assays, we performed genome-wide transcriptomic studies, as recently described [44]. We employed RNA sequencing (RNAseq) on cells transfected with either non-silencing scrambled control (SCR) or small interfering *SMAD3* (si*SMAD3*) RNAs to characterize genes and pathways that are regulated by this transcription factor in HCASMC. We identified 493 differentially expressed (DE) genes (FDR ≤ 0.05), as assessed with the DESeq algorithm [45]. Investigation of these DE genes with the Ingenuity Pathway Analysis (IPA) software (Qiagen) identified several overlapping canonical pathways, including “regulation of epithelial mesenchymal transition” (*p* = 1.15e-05), “axonal guidance signaling” (*p* = 1.17e-05), and “semaphorin signaling” (*p* = 1.36e-05) (Table 1). The highest degree of association with causal networks was identified for those regulated by the CAD associated CXCR4 cytokine (*p* = 3.32e-15) [6, 7], the chromatin regulating KAT2B (p300) lysine acetyltransferase (*p* = 3.55e-15), and the CAD associated soluble guanylate cyclase signaling molecule GUCY (*p* = 1.24e-14) [7]. IPA identified a high degree of enrichment for *SMAD3* knockdown DE genes among those associated with “cardiovascular disease” (*p* = 1.62e-4–8.37e-9) (Table 1, S1 Table), including those associated with vascular developmental syndromes such as “abnormal morphology of vasculature,” “abnormal morphology of blood vessels”, “abnormal morphology of artery,” as well as those associated with atherosclerotic vascular disease, “occlusion of blood vessel”, and “atherosclerosis”. These functional categories were enriched for genes in the endothelin signaling pathway, including CAD associated gene *EDNRA*, as well as *EDN1*, *EDNRB*, and *ECE*, smooth muscle cell differentiation factor *MYOCD* and vascular development factor *ANGPT1* (Fig 2).

**Table 1:** Network, pathway, disease, development and function, molecular function terms based on analysis of genes identified as significantly differentially regulated when si*SMAD3* treated HCASMC were analyzed with RNA sequencing.

**Top Pathways and Networks **	
**Canonical pathways–**Regulation of EMT, axonal guidance signaling, semaphorin signaling	
**Causal networks–**CXCR4, KAT2B, GUCY, DMP1	
**Cardiovascular Disease **	
***Functional Annotation***	**p*-Value***
abnormal morphology of vasculature	8.37E-09
congenital anomaly of cardiovascular system	7.37E-08
abnormal morphology of blood vessel	3.12E-07
abnormal morphology of artery	2.72E-06
hypertension	5.14E-06
disorder of blood pressure	6.49E-06
abnormal morphology of cardiovascular system	1.07E-05
occlusion of blood vessel	1.13E-05
atherosclerosis	1.81E-05
vaso-occlusion	2.62E-05
**Cardiovascular System Development and Function **	
***Functional Annotation***	**p*-Value***
development of vasculature	1.12E-18
angiogenesis	6.49E-18
vasculogenesis	1.62E-17
morphology of vasculature	4.85E-10
cell movement of endothelial cells	5.69E-10
morphology of vessel	6.36E-10
morphology of cardiovascular system	2.00E-09
morphology of blood vessel	5.57E-09
abnormal morphology of vasculature	8.37E-09
cardiogenesis	9.55E-09
migration of vascular endothelial cells	1.22E-07
morphology of artery	1.68E-07
abnormal morphology of blood vessel	3.12E-07
morphogenesis of cardiovascular system	2.49E-06
abnormal morphology of artery	2.72E-06
mean arterial pressure	5.42E-06
proliferation of vascular smooth muscle cells	6.81E-06
abnormal morphology of cardiovascular system	1.07E-05
contraction of blood vessel	7.96E-05
**Molecular and Cellular Functions**	
***Functional Annotations***	**p*-Value***
cell movement	1.67e-04–8.21e-17
gene expression	5.87e-07–2.82e-14
cell death and survival	1.19e-04–5.56e-12
cellular development	1.62e-04–6.08e-12
cellular growth and proliferation	1.62e-04–6.08e-12

In the highly relevant Physiological System Development and Function analysis in IPA, the top three terms identified for the *SMAD3* knockdown DE genes were identical to those identified in the similar analysis conducted with the CAD GWAS associated genes identified in a recent meta-analysis [7], “cardiovascular system development and function, “organismal development” and “organismal survival.” (S2 Table). While these category terms are quite broad, they suggest significant overlap between SMAD3 regulated genes and those identified in CAD associated loci. To rigorously test the significance of the overlap between the two gene lists, we employed the full GWAS catalog gene list as background and obtained a p-value of 0.000106 when employing the Fisher exact test. Cardiovascular system category terms (*p* = 1.12e-18) included “development of vasculature,” “angiogenesis,” and “vasculogenesis” (Table 1, S3 Table). Prominent genes in these categories included *SPRY4*, *FGF1*, and *HGF*, as well as various semaphorin and ephrin factors known to have roles in neuronal and vascular development [46–50]. Also, of interest were terms related to smooth muscle functions including “mean arterial pressure,” “proliferation of vascular smooth muscle cells”, and “contraction of blood vessel.” Top molecular and cellular functions included “cell movement” (*p* = 1.67e-04–8.21e-17), “gene expression” (*p* = 5.87e-07–2.82e-14), and “cell death and survival” (1.19e-04–5.56e-12) (Table 1, S4 Table).

Taking the 89 genes in the functional subcategory “vasculature development” in the Cardiovascular Development and Function category, we used well-curated molecular interactions in the IPA Knowledge Base to build a gene network (Fig 2, S5 Table). This subcategory was felt to be particularly pertinent to the molecular and cellular basis of CAD, based on the highly significant enrichment for such terms in the IPA analysis of the CAD GWAS meta-analysis genes [7], and the close match to terms in this category found here with *SMAD3* knockdown transcriptomic analysis. The interactions of the network DE genes were visualized with Cytoscape to highlight the mechanism of interaction between nodes and the various features of the relationship of each gene to the network as a whole (Fig 2). This network highlights the interaction of *SMAD3* with highly connected node genes that reflect integration of *SMAD3* with important vascular functions, including the endothelin pathway (*EDN1*, *EDNRA*, *EDNRB*, *ECE*), the *HGF-MET* signaling axis, *CXCL8* inflammatory pathway, and *RBPJ* component of Notch signaling.

Interestingly, many of the key vascular developmental genes were noted to also be regulated by TCF21 [34, 51], but in the opposite direction, including *EDNRA*, *EMP2*, *FGF7*, *ID1*, *ID3*, *IL24*, *ITGB8*, *SEMA3A*, *SEMA5A*, etc. Also, a number of matrix genes were differentially regulated by SMAD3 and TCF21 in opposite directions, including *COL1A1*, *COL1A2*, *COL3A1*, *COL5A2*, *THBS1*, and *ITGAV* among others. Some of these differences were visualized by mapping the *SMAD3* differential gene expression values onto the *TCF21* “cardiovascular disease” transcriptional network (S2 Fig) [34]. This comparison also identified some genes that are regulated in the same direction, including some matrix genes (*MMP2*, *MMP3*, *FBN1*) and vascular development genes (*SEMA3D*, *NRP1*, *ANGPT1*). A number of the TIMP and MMP genes that were differentially regulated by *SMAD3* and *TCF21*, and of interest in vascular disease processes, were further investigated by qRT-PCR. These studies documented differential regulation of *TIMP1*, *TIMP3* and *MMP10* by *SMAD3* expression (S3 Fig).

### ChIPseq localization of SMAD3 binding identifies loci encoding developmental and disease associated genes

**Fig 3:**
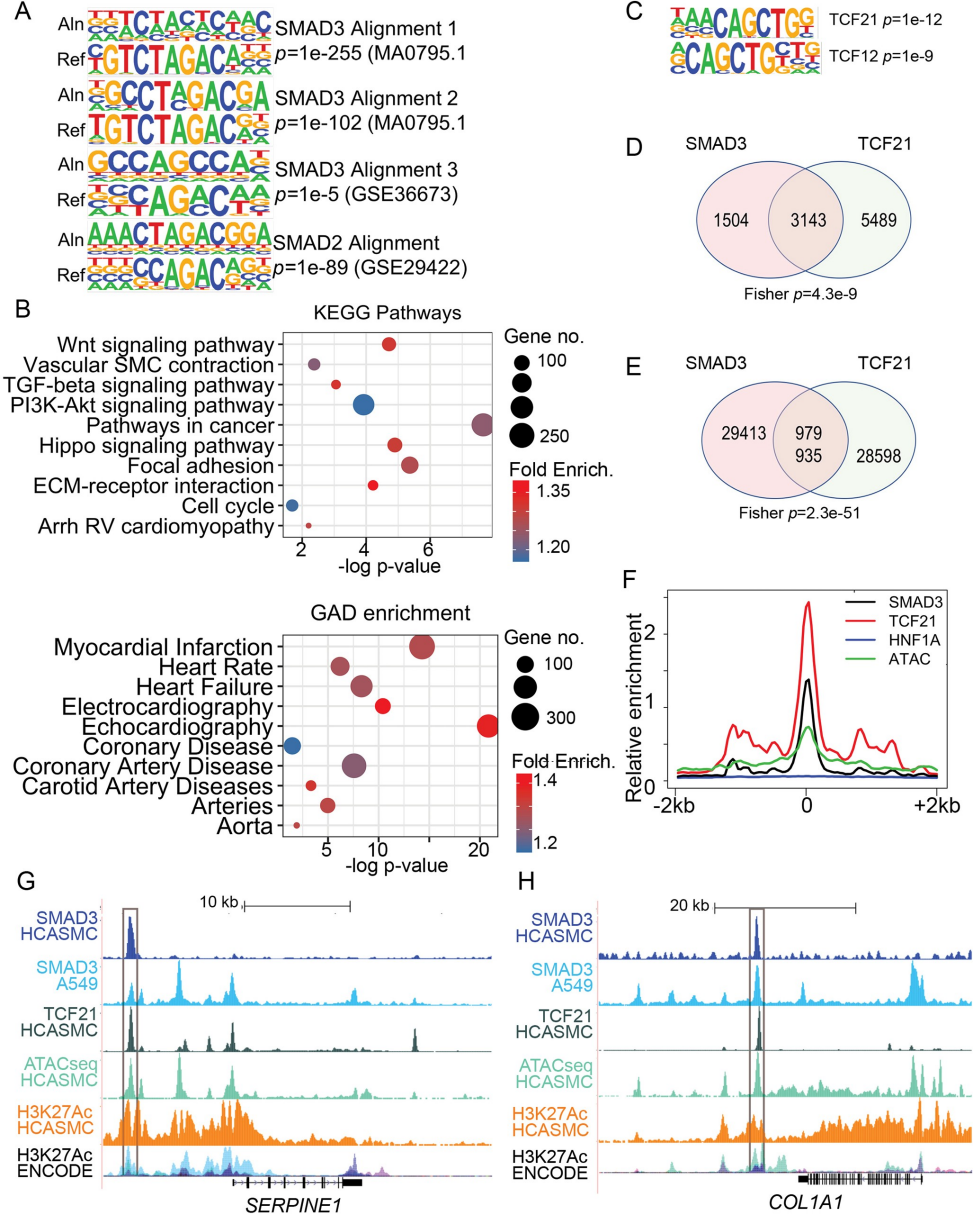
SMAD3 ChIPseq in HCASMC identifies targeted genes in developmental and disease pathways and regions of SMAD3 and TCF21 colocalization. A) SMAD3 motifs from HOMER *de novo* analysis of SMAD3 peaks. Aln sequence is the aligned HCASMC SMAD3 peak sequence and Ref indicates the reference binding sequence. B) DAVID Gene Ontology analysis of all SMAD3 target genes identified by GREAT in “basal plus extension” mode, including KEGG pathways and GAD disease enrichment analysis. C) Homer “known” motif analysis of intersected SMAD3 and TCF21 peaks identified the TCF21 binding sequence as the top motif, and also found enrichment of the binding sequence for the TCF21 partner TCF12. D) Venn diagram showing the overlapped gene number between filtered SMAD3 peak (fold change>5, -logQ>10) target genes and filtered TCF21 peak (fold change>15, -logQ>200) target genes, as identified with GREAT. E) Venn diagram showing the number of filtered TCF21 peaks (fold change>5, -logQ>60) located within the intervals of all SMAD3 peaks extended by ±1 kilobase. F) Overlapping peaks identified in E were extended ±1 kb from SMAD3 peak summits, and a density plot created for enrichment levels of SMAD3, TCF21, and control transcription factor HNF1A, along with HCASMC ATACseq open chromatin. G) Pattern of SMAD3 and TCF21 overlapping binding at the human *SERPINE1* locus. In addition to SMAD3 and TCF21 ChIPseq data, also shown are additional HCASMC genomic profiling, including ATACseq mapping of open chromatin and H3K27Ac ChIPseq mapping of enhancer marks. Published SMAD3 ChIPseq data from A549 cells [52], and ENCODE H3K27Ac data are shown as well. H) Similar SMAD3 ChIPseq data in the *COL1A1* locus.

To identify genes that are directly regulated by SMAD3, and to link this CAD gene with other genes that are associated with CAD, we performed chromatin immunoprecipitation sequencing (ChIPseq) with cultured HCASMC. SMAD3 ChIPseq identified 30,292 total binding sites in the HCASMC genome. The ChIPseq findings in HCASMC were validated by ChIP-PCR of representative well documented SMAD3 target loci, *TAGLN*, *CNN1*, *COL1A1*, and *SERPINE1*, verifying SMAD3 binding that was increased with TGFβ1 stimulation (S4A Fig). For comparison to the HCASMC data, we used the same analysis pipeline and analyzed the published ChIPseq data obtained for the A549 lung cancer cell line [52]. Interestingly, intersection of the 24,587 A549 and HCASMC peaks, requiring overlap of at least one basepair identified sharing of only 1076 of the HCASMC peaks and 1393 of the A549 peaks. Increasing the binding site by 1 kb on either side of the peak only increased the number of overlapping HCASMC SMAD3 and A549 SMAD3 peaks to 1826 and 2303 respectively. Further, use of the HOMER “de novo” algorithm identified several related SMAD3 motifs in the HCASMC data which were specific variants of the three well characterized binding sequences (Fig 3A) [52]. Taken together these analyses suggest that SMAD3 binds a unique repertoire of regions in the HCASMC genome, consistent with the unique integral role of TGFβ in the transcriptional regulation of the phenotype of this cellular lineage [30, 53, 54].

SMAD3 peaks were assigned to genes with the Genomic Regions Enrichment of Annotations Tool (GREAT) [55], and this collection of target genes were employed in gene ontology analysis using DAVID (Fig 3B, S4B Fig). Terms identified by Biological Process analysis included highly relevant significant terms including “vasculogenesis”, “transcription from RNA pol II promoter”, and “regulation of cell differentiation.” Significant KEGG pathways included “Wnt signaling,” “vascular smooth muscle contraction,” and “TGFβ signaling pathway” terms. Disease enrichment terms included a number of significantly relevant terms highlighting target gene association with CAD, including “myocardial infarction,” coronary disease,” and “coronary artery disease.” Molecular function pathways were largely related to transcriptional regulation and SMAD-DNA binding (S4B Fig).

### SMAD3 target regions also bind TCF21

Given the marked functional dichotomy between SMAD3 and TCF21, which promotes a de-differentiation program in HCASMC [34], we were interested to investigate possible interactions that might reflect coordinated regulation of HCASMC phenotype, and thus disease risk. First, we intersected the SMAD3 ChIPseq and our standard TCF21 HCASMC ChIPseq datasets (see Methods) [56] to identify regions of the genome that mediate binding of both transcription factors. Scanning the overlapped SMAD3 peaks for known binding motifs with the HOMER “known” algorithm, we found the CAGCTG TCF21 binding sequence to be the most highly ranked (*p* = 1e-12), suggesting that TCF21 binds in proximity to SMAD3 in a significant number of colocalizing loci (Fig 3C). Also, the binding sequence for TCF12, a TCF21 obligate heterodimer was also enriched in these SMAD3 peaks (*p* = 1e-9). Further, we investigated characteristics of the genomic overlap patterns of the two ChIPseq datasets to better understand how they might be clustered on a whole genome level. First, we investigated the possible overlap of binding at a gene level. For this analysis, we intersected a restricted set of data for both SMAD3 (fold change>5, -logQ>10) and TCF21 (fold change>15, -logQ>200), assigning genes to peaks with GREAT. With this approach, we found 4,647 genes were assigned to the SMAD3 peaks, with 3,143 or 68% of these genes also identified among the 8632 total genes assigned to TCF21 peaks (Fig 3D).

Further, by intersecting the full SMAD3 dataset and the standard TCF21 dataset, 979 of 30,392 SMAD3 peaks were found to overlap 935 of 29,533 TCF21 ChIPseq peaks (Fig 3E). The apparent disparity between the results from these two analyses (Fig 3D and 3E) was due to the fact that although the peaks reside in the same loci, and therefore assigned to the same genes, they are distant enough to not share basepairs except for 979 SMAD3 peaks and 935 TCF21 peaks. We further investigated the colocalization of SMAD3 and TCF21 binding in these ~1000 loci with density plots for SMAD3, TCF21, and the negative control TF HNF1A, along with HCASMC ATACseq regions of open chromatin in HCASMC (Fig 3F). This analysis revealed an overlap of binding sites for SMAD3 and TCF21 in ATACseq open chromatin regions, compared to HNF1A. Further, we investigated the relationship between SMAD3 and TCF21 binding and epigenetic features in restricted areas of colocalization for loci encoding genes of interest in disease pathophysiology (Fig 3G and 3H). In loci encoding the *SERPINE1* and *COL1A1* genes, we identified overlap of SMAD3 and TCF21 peaks, in regions of HCASMC open chromatin as identified with ATACseq. Further, there was colocalization with the H3K27Ac histone mark as identified in HCASMC and in ENCODE samples. For these two genes, there was overlap of HCASMC SMAD3 peaks with some of those identified by ChIPseq in A549 epidermal lung cancer cells, although there were also significant differences in the binding patterns.

Finally, to investigate the biological pathways represented by the genes in those loci where SMAD3 and TCF21 co-localize, we assigned genes to this set of peaks with GREAT and performed GO analysis. This analysis identified terms quite similar to those found for the SMAD3 ChIPseq dataset alone (Fig 3B, S4C Fig). However, the overall number of genes identified for each term was smaller and the *p*-values were in general smaller than the results with the full SMAD3 targeted gene list. For example, in the KEGG pathways, there was a significantly smaller *p-*value for “pathways in cancer” and “Hippo signaling,” and in the Biological Processes category for “cell migration” and “transcription from RNA pol II promoter.” Surprisingly, in the Disease Enrichment category CAD related terms “myocardial infarction” *p*-value were less significant while “coronary artery disease” and other related term *p*-values were unchanged.

### SMAD3 binding to DNA and activation of transcription are inhibited by TCF21

**Fig 4:**
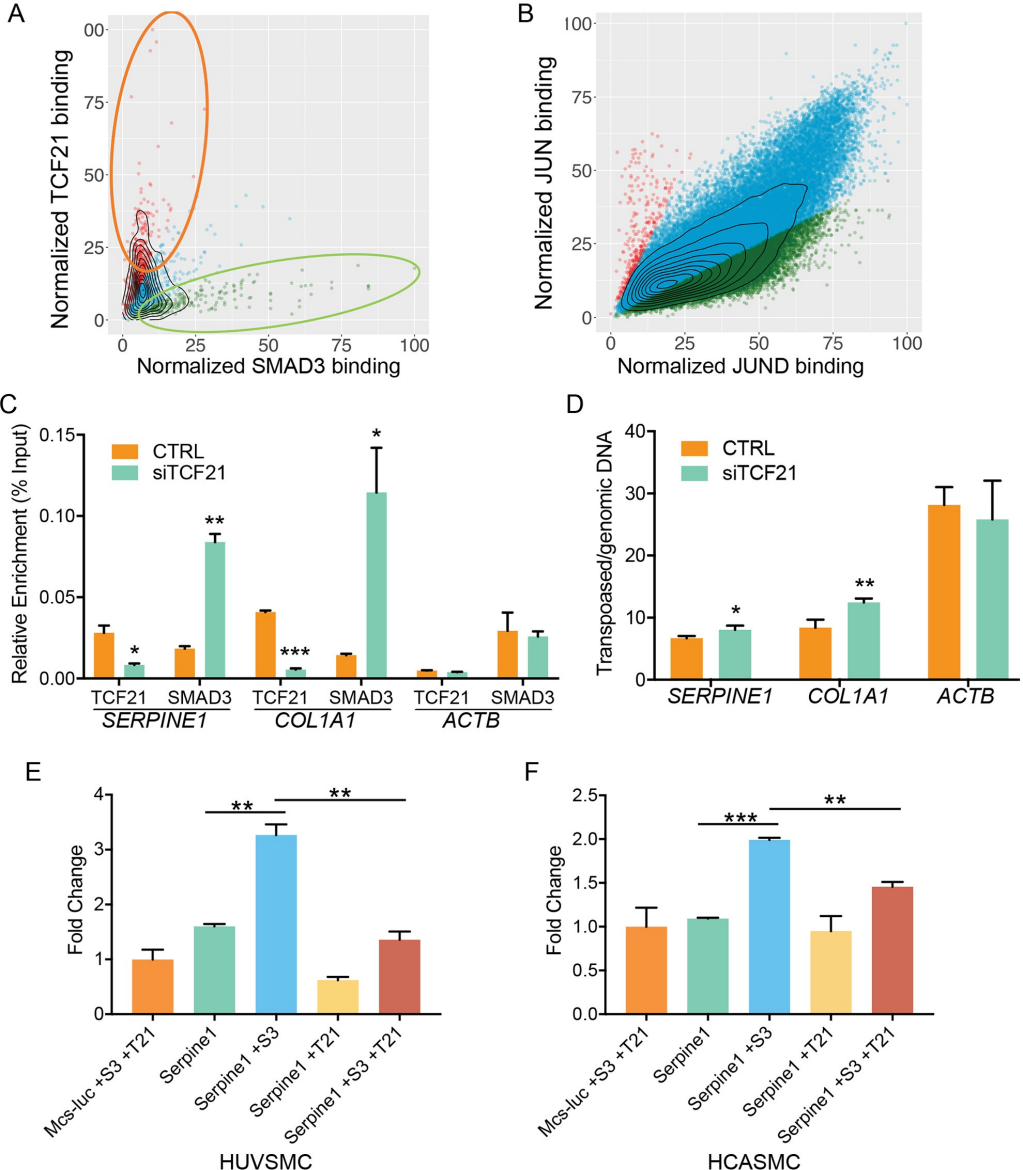
SMAD3 and TCF21 have opposing binding behavior and transcriptional regulatory functions in HCASMC. A) SMAD3 and TCF21 joint binding regions were analyzed for degree occupancy by each factor. DNA occupancy was assessed by comparing peak reads normalized to background reads and this variable graphed. More than half of the colocalized binding regions were biased for either SMAD3 or TCF21 binding, with a 2-fold greater number of normalized reads for one or the other factor. B) To serve as a control for SMAD3 and TCF21 binding patterns, we characterized binding of AP1 heterodimer transcription factors JUN and JUND. Compared to the SMAD3 and TCF21 pattern, there is equivalent binding at the majority of AP1 target sites in the genome. C) SMAD3 binding at the *SERPINE1* and *COL1A1* loci where it colocalizes with TCF21 was assessed by ChIP-PCR in HCASMC. Knockdown of *TCF21* mRNA levels by specific siRNA was associated with increased binding of SMAD3 compared to cells transfected with scrambled siRNA (SCR). D) Local chromatin accessibility was evaluated at the *SERPINE1* and *COL1A1* loci with the assay for transposase-accessible chromatin coupled with quantitative PCR (ATAC-PCR), in HCASMC with knockdown by si*TCF21*. E) Reporter gene transfection studies evaluated relative transcriptional activity of SMAD3 and TCF21 at a *SERPINE1* enhancer region, in an established human umbilical vein smooth muscle cell line (HUVSMC). F) Identical experiments were conducted in primary cultured HCASMC.

Given the opposite effects of SMAD3 and the CAD associated transcription factor TCF21 on the differentiation state of HCASMC [34], and evidence that these two transcription factors colocalize in a number of shared binding regions of the genome where they may regulate the same genes, we were interested to determine if there is a functional relationship in shared loci. To investigate binding patterns suggestive of interactions between SMAD3 and TCF21 at the level of protein binding to DNA, we analyzed the relative binding of each factor by normalizing the number of reads in the peaks to background counts in the region (see Methods). We focused on those binding sites which showed a greater than two-fold difference in normalized read counts, as a measure of relative binding (Fig 4A and 4B). For the directly overlapping ChIPseq binding sites for SMAD3 and TCF21, more than half showed a 2-fold discrepancy in binding. Out of 583 biased binding sites, 358 (358/583, 61%) showed higher relative binding of TCF21 and 225 (225/583, 39%) showed higher relative binding for SMAD3 (Fig 4A). This pattern of SMAD3 compared to TCF21 binding at shared loci is quite distinct from the expected pattern for factors that bind with equal affinity, as shown for JUN and JUND binding, where only a small percentage of the binding was at sites with a greater than 2-fold difference (Fig 4B). These findings suggested that one of these TFs might inhibit binding of the other at these shared loci, a mutual inhibitory interaction, or opposing responses to external signaling pathways.

To investigate the possibility that TCF21 inhibits SMAD3 binding at these loci, we performed ChIP-PCR at two representative loci, encoding the *SERPINE1* and *COL1A1* genes. PCR primers were designed to amplify regions where both SMAD3 and TCF21 bind in areas of open chromatin and active histone mark configuration (Fig 3G and 3H). As a control, ChIP-PCR was also conducted for the *ACTB* gene at a SMAD3 binding site where there is no evidence of TCF21 binding. HCASMC treated with scrambled, control, siRNA showed significant enrichment for SMAD3 binding as expected at loci identified by ChIPseq studies. SMAD3 binding increased significantly at the *SERPINE1* (0.018 vs 0.084, p<0.01) and *Col1A1* loci (0.014 vs 0.114, p<0.05) loci with effective knockdown of *TCF21* expression (Fig 4C). Employing the assay for transposase-accessible chromatin coupled with quantitative PCR (ATAC-PCR), we further investigated whether TCF21 modulation of SMAD3 binding might be associated with changes in local chromatin accessibility. The ATAC-PCR studies showed that knockdown of *TCF21* produced significantly increased accessibility at the SMAD3 binding site in both *SERPINE1* (6.67 vs. 8.02, p<0.05) and *COL1A1* (8.40 vs 12.41, p<0.01) (Fig 4D). These data are consistent with TCF21 indirectly inhibiting SMAD3 binding in regions of colocalization through an epigenetic mechanism affecting chromatin accessibility.

Finally, to investigate possible direct interactions between SMAD3 and TCF21 regulation of gene expression, we performed reporter gene transfection studies with an enhancer region in the *SERPINE1* gene where putative binding sites for these TFs are separated by 78 basepairs (Fig 3G). This region ~9kb upstream of the transcription start site of the human *SERPINE1* gene was cloned into a reporter plasmid, and co-transfected into a human umbilical vein smooth muscle cell line (HUVSMC) and primary cultured HCASMC, with expression plasmids for *SMAD3* and/or *TCF21* (Fig 4E and 4F). The enhancer alone increased expression of the basal reporter, and transcription was further increased with *SMAD3* over-expression. *TCF21* transfection significantly inhibited expression of the basal enhancer, and mitigated the increase that was seen with SMAD3 over-expression in both HUVSMC (3.27 vs 1.30, p<0.01) and HCASMC (2.00 vs 1.46, p<0.01). Thus, at this enhancer where SMAD3 and TCF21 binding colocalize, TCF21 can significantly inhibit the transcriptional effect of SMAD3. Since the reporter constructs were not integrated into the chromatin where epigenetic modification by TCF21 could affect SMAD3 function, these data suggest a separate mechanism, with these TFs producing independent opposing effects on the basal transcription apparatus, or possibly TCF21 blocking SMAD3 binding through direct protein-protein interaction.

### CAD association and HCASMC eQTL data indicate that SMAD3 promotes CAD risk

**Fig 5:**
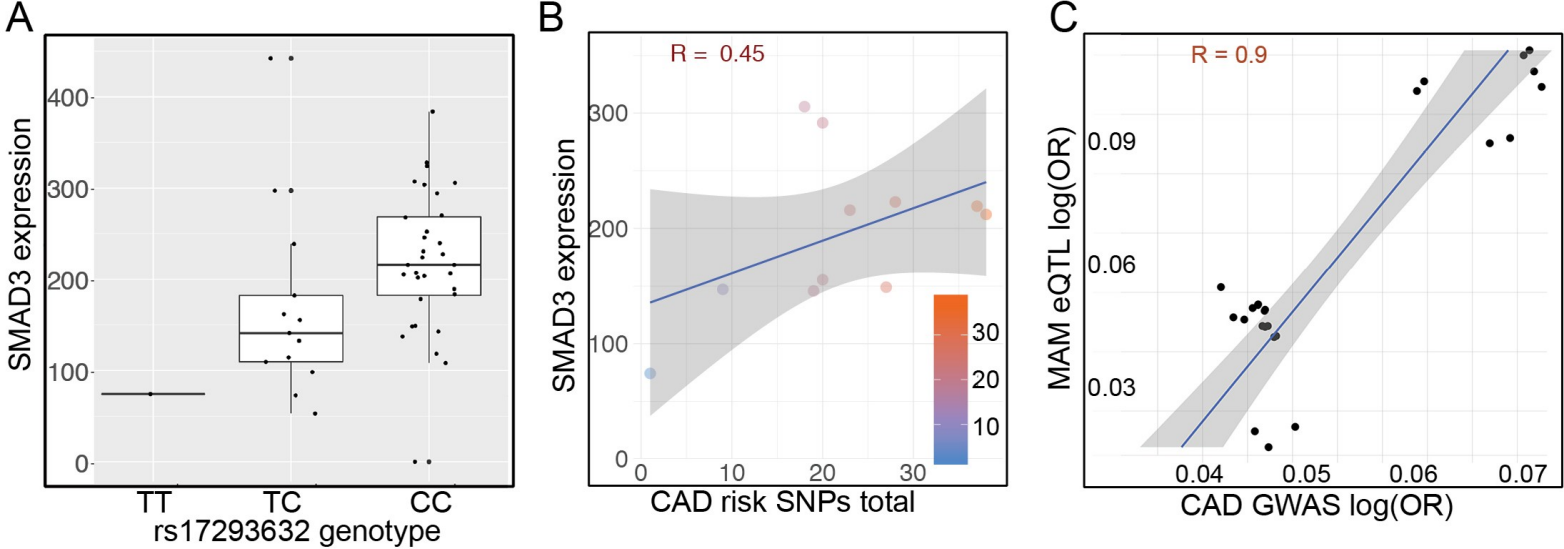
*SMAD3* expression is directly correlated with disease risk. A) HCASMC eQTL data was employed to investigate the directionality of associated disease variation on *SMAD3* expression in a highly disease relevant cellular model. The disease risk C allele genotype at rs17293632 was associated with increased *SMAD3* expression. This genotype effect on expression was significant at *p*<0.05 employing a linear regression model. B) To investigate all variation that might contribute to differential expression of *SMAD3* and thus disease risk, we studied CAD associated SNPs from the latest CARDIOGRAM+C4D meta-analysis [7] that were associated with CAD at *p*<1.0e-6 and that were located up to 100kb away from the *SMAD3* gene. This regression analysis with HCASMC eQTL data had a Perrson correlation coefficient of 0.45 and was nominally significant at *p* = 0.08, suggesting that *SMAD3* expression increases with a greater number of cis-risk alleles as per an additive model and further implicate *SMAD3* expression and function in disease risk in this cell type. C) All CAD associated SNPs at *p*<1.0e-4 from Nelson et al. [7] that were identified in the *cis*-eQTL summary results, having some association with *SMAD3* expression levels in artery tissues (internal mammary artery; MAM) in individuals with CAD from the STARNET study were plotted relative to each other based on their respective effect sizes (log OR) [60]. Pearson correlation coefficient R = 0.894, *p* = 3.76e-9.

While there has been much interest and study of the role of the TGFβ pathway in vascular disease pathophysiology, there remains much debate regarding the direction of effect for the pathway and specifically SMAD3 [19, 20, 57, 58]. Limited public expression quantitative trait loci (eQTL) datasets have suggested that *SMAD3* expression is associated with disease risk [11, 21]. We have extended these studies to investigate the causality and directionality of the *SMAD3* gene in CAD with three additional strategies. First, we assessed gene expression at the putative causal variant rs17293632 with disease relevant eQTL data from human coronary artery smooth muscle cells [59], and found that the risk C allele increases expression of *SMAD3* (*p*<0.05), indicating *SMAD3* is a CAD promoting transcription factor and suggesting that this gene is active in this cell type *in vivo* (Fig 5A).

In a second approach employing the HCASMC eQTL data, we assumed an additive model of CAD risk for alleles located in the *SMAD3* locus. For this analysis, we selected CAD associated SNPs from the latest CARDIOGRAM+C4D meta-analysis that were associated with CAD at a *p-*value cutoff of 1.0e-6 and that were located within 100kb away from the start and end of the composite *SMAD3* gene [7]. To improve the correlation in the local regions of strong linkage disequilibrium we used an algorithm (see Methods) to resolve the local haplotype structure in each HCASMC sample and average expression on those samples that possess identical haplotype profiles in the tested region. This reduced the variability by eliminating the component of the variance that arises from inter-individual variability and technical issues. By employing this approach, the regression coefficient was 0.45, with a nominally significant *p*-value of 0.08, suggesting that *SMAD3* expression increases with a greater number of cis-acting risk alleles as per an additive model (Fig 5B).

Finally, we have investigated the correlation between allelic risk and gene expression in mammary artery (MAM) tissue samples from individuals with coronary artery disease in the Stockholm-Tartu Atherosclerosis Reverse Networks Engineering Task study (STARNET) [60] (Fig 5C). We observed a strong positive correlation (r = 0.894; *p* = 3.76e-09) for the effect sizes (log OR) of CAD associated variants (*p*<1e-04) with those also associated with *SMAD3* gene expression (*cis*-eQTLs) in STARNET. Notably we did not observe a similar trend in atherosclerotic aortic tissues, in which *TCF21* was identified as a strong *cis*-eQTL gene. These findings leveraging natural genetic variation from GWAS and SMC-enriched artery tissues from CAD individuals further implicate *SMAD3* as a pro-atherosclerotic gene, consistent with findings in HCASMC.

## Discussion

Having recently established the likely causality of *SMAD3* for CAD [11], the overall goal of the work reported here was to determine the mechanism and direction of effect for this gene, and thus integrate it into a causal framework that regulates disease pathophysiology. We have demonstrated with various genetic and genomic approaches that a significant portion of the genetic risk for CAD resides in the smooth muscle cell lineage and that the TGFβ pathway in particular regulates genomic features in disease loci, and have thus focused here on HCASMC [11, 59]. These studies confirm previous work in other SMC models that SMAD3 promotes a differentiation program in these cells, as evidenced by upregulation of lineage markers [22, 61, 62]. Further, we have shown that SMAD3 binds in regions of the genome that also harbor TCF21 binding sites, and provide several lines of evidence that point to antagonistic effects between DNA binding and transcriptional action of these two TFs, primarily for TCF21 inhibition of SMAD3. These data suggest that TCF21 may mediate this effect through modifying the epigenome and also possibly directly inhibiting SMAD3 binding to DNA. Finally, we have provided the most compelling evidence to date, employing eQTL data from HCASMC and vascular tissues, that expression of SMAD3 is directly causal for CAD. We propose that the pro-differentiation function of SMAD3 inhibits HCASMC dedifferentiation, phenotypic modulation, of these cells as they respond to vascular stress and expand to stabilize the plaque, and that SMAD3 function is directly opposed at the transcriptional level by the disease protective expression of TCF21, which promotes dedifferentiation and phenotypic modulation.

Our results showing SMAD3 promotion of HCASMC marker expression and proliferation are consistent with previous studies investigating TGFβ signaling in other types of SMCs [24, 61], and are consistent with a pro-differentiation paradigm. Although previous in vitro studies have shown a migratory effect of TGFβ signaling [43], our data showing that SMAD3 can promote HCASMC migration would seem at odds with the marker gene and proliferation effects, and raise the question of whether SMAD3 is truly promoting differentiation of HCASMC or merely acting as a transcriptional regulator in these experiments. At a molecular level, it is known that SMAD3 binds the SMC transcription factor myocardin to promote this transcriptional program [30]. Myocardin is widely regarded as a lineage determining factor, fundamentally specifying the SMC fate, and thus serving as more than a transcription factor that activates contractile marker expression [63]. Also, it is well known that TGFβ signaling has a role in embryonic vascular development, specifically promoting induction of SMC markers in mesodermal cells that become phenotypic SMC, and also promoting their migration to the forming vascular structure [22]. Thus, there is precedent for SMAD3 signaling jointly promoting SMC differentiation and migration. Further, for these studies, HCASMC were evaluated in media containing a number of growth factors and cytokines found in serum and growth additives. Whichever factors promote migration in this setting may not be functional in vivo in the disease setting. Migratory activity would seem to be under the control of a number of factors with competing programs. In the context of SMAD3 effects on disease risk, pro-differentiation and inhibited cell division are at odds with the protective effects of TCF21 in the vessel wall and thus likely disease promoting, while increased migration is also promoted by TCF21 and likely protective. Fortunately, this conundrum is resolved by human genetic data that clearly indicate that expression of SMAD3 increases risk for human CAD, possibly by providing a stimulus for SMC to remain differentiated and oppose phenotypic modulation that appears to be protective.

The TGFβ pathway has been linked to a number of vascular diseases. These include syndromic diseases associated with aortic aneurysms, including Marfan’s and Loeys Dietz Syndromes (LDS), due to mutations in TGFβ signaling genes *FBN1*, *TGFBR1*, *TGFBR2*, *TGFB2*, and *TGFB3* [64, 65]. Also, *SMAD3* mutations have been linked to the syndromic disease Aneurysms Osteoarthritis Syndrome [29]. In general, these aortopathies are characterized by large vessel aneurysms that primarily result from loss of SMC and related structural matrix components [29]. A paradox in the field is that disease mutations that should be amorphic, including *SMAD3* mutations associated with aortopathy, actually produce increased TGFβ signaling as the mechanism of effect [65]. The relationship between the TGFβ pathway and common complex vascular diseases such as CAD has been more difficult to establish. The literature is replete with reports of in vitro and in vivo model systems studies of this pathway in vascular disease, but there have been much debate regarding the directionality of effect for TGFβ signaling, and SMAD3 function in particular, on disease initiation and progression [19, 20, 57, 58]. Interest in the role of TGFβ signaling in CAD has been renewed because of the CAD GWAS association of numerous loci that harbor TGFβ signaling molecules [10, 11].

In addition to *SMAD3*, a striking recent finding in GWAS meta-analyses has been the identification of the *TGFB1* locus as a CAD associated region of the genome [6–8]. Although *TGFB1* has not been identified as the causal gene in this locus through mechanistic studies, and there are not highly informative eQTLs in vascular cells or tissues, algorithms that integrate available gene expression and CAD association data, Summary data-based Mendelian Randomization [66] and MetaXcan [67], provide compelling support for the causality of *TGFB1*. These data suggest that *TGFB1* expression promotes CAD risk, which is consistent with the fact that canonical TGFβ1 signaling occurs primarily through the risk-promoting SMAD3 pathway. While much remains to be learned regarding the role of TGFβ family members in vascular disease, mutations associated with Mendelian aortopathies as well as common variation associated with CAD, appear to result from increased TGFβ signaling.

A number of previous studies have provided evidence that TGFβ signaling promotes the differentiated phenotype, and that SMAD3 is involved at a molecular level in this process through direct interaction with the lineage determining factor myocardin (*MYOCD*) [30]. However, given the unique embryonic origin, the singular phenotypic characteristics of coronary artery smooth muscle cells, and the importance of this question in the context of recent GWAS studies linking SMAD3 to CAD risk, we sought to investigate in detail how SMAD3 affects the cell state of HCASMC, and how it might interact at a molecular level with other factors that are involved in the relationship between SMC phenotype and CAD. In vitro studies provided clear evidence that SMAD3 expression promotes expression of differentiation markers, and inhibits proliferation of HCASMC, and this phenotype contrasts to that promoted by CAD associated factor TCF21 which inhibits HCASMC differentiation [34]. ChIPseq data indicated that the majority of SMAD3 binding loci also bind TCF21 (Fig 3D), and that TCF21 binds adjacent to SMAD3 in ~3% of loci (Fig 3E). Detailed analysis of the normalized read depth at binding sites in these loci revealed an inverse correlation of SMAD3 and TCF21 binding, suggesting that these TFs might inhibit the binding of each other in these regions of the genome. This hypothesis was supported by ChIP-PCR studies in TCF21 depleted HCASMC which showed greater SMAD3 binding in two loci that harbor adjacent colocalization of these two factors. The observed interaction was shown to be due in part to local regulation of chromatin accessibility by TCF21. It is known that TCF21 recruits HDACs, and that SMAD3 recruits coactivators such as histone acetyltransferases (HATs) p300 and CREB binding protein (CBP), and may also recruit primarily repressive histone deacetylases (HDACs) [68, 69]. Further, reporter gene transfection studies with a *SERPINE1* enhancer region that binds both SMAD3 and TCF21 revealed that *TCF21* expression inhibited the SMAD3 positive effect on transcription, an effect that could represent independent effects mediated through individual TF binding sites, or could be due to direct interaction of these TFs affecting the binding of one another. Taken together, these data suggest two possible mechanisms by which TCF21 affects the transcriptional function of SMAD3, through modification of the epigenome in regions where they colocalize, and also that TCF21 can directly affect/inhibit SMAD3 binding and transcriptional promotion of the SMC differentiation program, across small genomic distances as has been described for other TF interactions [70].

**Fig 6:**
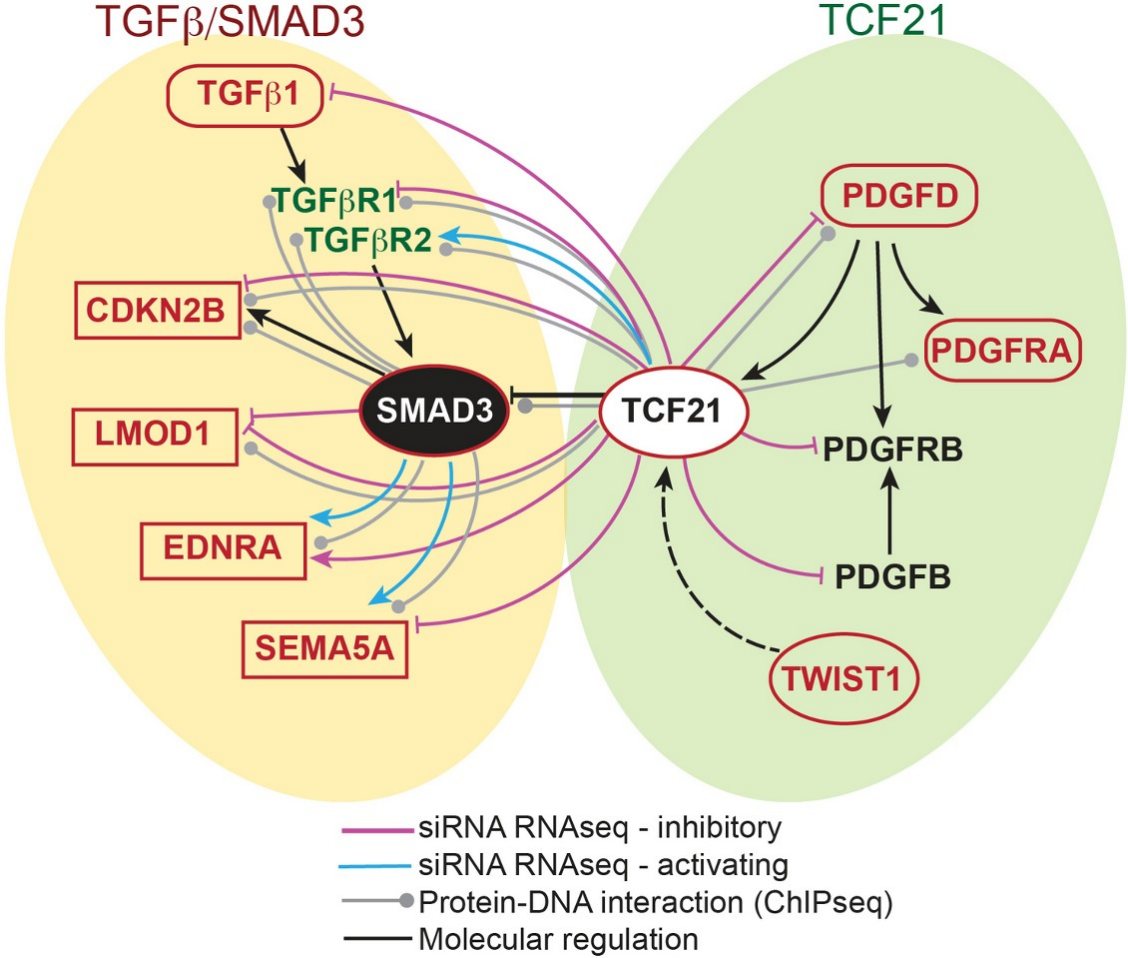
Network of CAD associated genes that may regulate HCASMC phenotype as a mechanism of disease risk. Genes in red text reside in replicated CAD associated loci, with genomic and/or functional data supporting their causality [73, 75] [6, 7, 11, 21, 32–34, 56]. Gene symbols in ellipses indicate CAD associated transcription factors. HCASMC ChIPseq data presented here or previously published provide evidence of direct transcriptional regulation and RNAseq data provides evidence of directionality [34, 56]. These and other data reveal a pervasive role of the TGFβ pathway in CAD causality [10], and also suggest that linked TCF21 and PDGF signaling is an important component of disease risk.

Given the work reported here for the SMAD3 transcription factor, and growing information for GWAS loci genes that are expressed and functional in HCASMC [33, 71–73]it is possible to begin to establish a disease related transcriptional network for CAD in this cell type (Fig 6). Findings from these RNAseq and ChIPseq studies show that a number of SMAD3 downstream targets are validated or leading candidate causal genes in CAD associated loci, including *CDKN2B*, *LMOD1*, *EDNRA*, and *SEMA5A*. SMAD3 binds the loci for two of its upstream receptors, TGFβR1 and TGFβR2, but RNAseq data did not show their differential regulation in cultured HCASMC. Interestingly, these genes are also targets of TCF21, with the direction of effect primarily being the opposite of SMAD3, primarily inhibitory. In addition to SMAD3, TCF21 binds and regulates numerous TGFβ pathway factor genes, including *TGFB1*, *TGFBR1* and *TGFBR2*. Although not directly related to TGFβ signaling, TCF21 inhibits expression of the CAD *PDGFD* gene, as well as *PDGFB* and *PDGFRB* receptor genes. The putative SMAD3-TCF21 interactions are consistent with data from various types of experiments presented here which indicate that TCF21 has an inhibitory role toward SMAD3 regulation of gene expression. While directionality of disease effect is in general difficult to establish, compelling data from HCASMC and vascular eQTL data presented here suggest that expression of *SMAD3* is directly correlated to disease risk, and importantly these findings are opposite to those indicating that expression of *TCF21* has a protective role [33, 59]. Further, given that expression of *SMAD3* appears to promote disease risk along with HCASMC differentiation, and *TCF21* promotes de-differentiation of this cell type and inhibits disease risk, these findings together argue that the process of phenotypic modulation is protective toward disease risk [33, 34, 74].

A limitation of the work reported here is the absence of correlation with appropriate in vivo experiments in appropriate animal models, such as genetic mouse models of atherosclerosis. For instance, combination of conditional deletion in the SMC lineage with concomitant lineage tracing of the targeted cells would be predicted to show that loss of SMAD3 expression would lead to increased exit of SMC from the media. Also, a concomitant increase in the number of SMC-derived cells in the plaque, and possibly at the fibrous cap, would be anticipated. Correlation of histological findings in diseased and normal human tissues samples from individuals of known genotype could provide compelling corroborative data regarding the molecular mechanism and direction of effect. These and other mechanistic studies are expected to support the human genomic and genetic data derived from experiments reported here.

## Materials and methods

### Primary cell culture and reagents

Primary human coronary artery smooth muscle cells (HCASMCs) were purchased from three different manufacturers, PromoCell, Lonza and Cell Applications at passage 2 and were cultured in smooth muscle cell basal media along with hEGF, insulin, hFGF-B and fetal bovine serum (FBS) (Lonza # CC-3182) according to the manufacturer’s instructions. HCASMCs between passages 5–8 were used for all the experiments. For the ChIPseq and ChIP-PCR studies, cells were serum starved for 48 hrs and then treated with 5 ng/ml human recombinant TGFβ1 (R&D Systems) for 6 hrs before crosslinking.

### SMAD3 knockdown and overexpression

*SMAD3* (s8401 and s8402) silencer select siRNAs were purchased from Life Technologies. siRNA transfection was performed using Lipofectamine RNAiMAX (Life Technologies). For each well treated with the *SMAD3* siRNA or scrambled control (Life technologies, #4390843), the final concentration was 20 nM. HCASMCs were seeded in 6 well plates and grown to 75% confluence before siRNA transfection. HCASMCs were transfected with the *SMAD3* siRNA or scrambled control for 12 hours and subsequently collected and processed for RNA isolation after 48 hrs of transfection using the RNeasy kit (Qiagen). For the *SMAD3* overexpression study, HCASMCs were transduced with 5ug of pRK5F-*SMAD3* cDNA (Addgene plasmid# 12625) or control pCDNA3.1 DNA (ThermoFisher Scientific, plasmid# V79020) using the Amaxa Basic Nucleofector kit for primary mammalian smooth muscle cells (Lonza #VPI-1004) at a density of 1x10^6^ cells per 100 μL sample using Nucleofector Program U-025. Cells were changed to medium with supplements 24hrs after transfection and cultured for an additional 48 hrs. The transduction efficiency was assessed by transducing HCASMCs with 2 μg of pmax-GFP cDNA and quantifying the percentage of GFP positive cells by quantitative fluorescence microscopy.

### In vitro cellular assays

For evaluation of the effect of SMAD3 on HCASMC lineage marker expression, cells were subjected to knockdown or overexpression of *SMAD3* as outlined above, and gene expression quantitated as below by qRT-PCR and western blotting. For quantitative immunofluorescence assay of marker expression, adherent HCASMCs were fixed with 4% paraformaldehyde (PFA) for 20 minutes, permeabilized with 0.5% Triton-X 100/PBS for 10 minutes and blocked with 3% BSA/PBS for 60 minutes at room temperature. Primary antibody incubations were performed at 4°C overnight followed by incubation with an Alexa Fluor-tagged secondary antibody for 60 minutes at room temperature. Nuclei were counterstained with Hoechst (1 μg/ml, Life Technologies). Images were acquired with a Zeiss Axioplan 2 microscope using the LASX software.

Migratory effects of SMAD3 expression were evaluated with a gap closure assay. The gap closure assay (Cell Biolabs #CBA-126) was conducted according to the manufacturer’s protocol. Briefly, 10,000 lentivirus transduced cells were seeded per well and let attach overnight. After gel removal 3 wells per condition were directly stained with crystal violet and imaged while the remaining 9 wells per condition were incubated for 12h before crystal violet staining. The covered area per well was quantified using ImageJ v1.47.

Proliferation of HCASMC with *SMAD3* knockdown and over-expression was evaluated with an in vitro EdU assay (Thermo-Fisher). HCASMC were serum starved for 24 hours. Following starvation, the cells were exposed to serum for 24 hrs, with treatment during the last 3 hrs of this period with EdU from the Click-iT EdU Alexa Fluor 488 Imaging Kit (Life technologies, Carlsbad, CA; Cat# C10377) at a concentration of 20uM. The cells were incubated with EdU for 3 hours, then fixed and permeabilized using 4% PFA and 0.5% Triton-X in PBS, respectively. This was followed by incubation with Click-iT reaction cocktail, including CuSO4 and Alexa Fluor Azide, and then with nuclear staining with DAPI solution. Using a Leica inverted microscope, the number of total nuclei, and the number of co-stained nuclei were counted using the ImageJ (NIH) software on ten consecutive 10x fields for each condition.

### RNA isolation and qRT-PCR

RNA for all samples was extracted using the RNAeasy mini kit (Qiagen). HCASMC RNA (500 ng) were reverse transcribed using the High capacity RNA-to-cDNA Synthesis kit (Applied Biosystems). Quantitative PCR of the cDNA samples was performed on a ViiA7 Real-Time PCR system (Applied Biosystems) and gene expression levels were measured using SYBR green assays using custom designed primers and normalized to PBGD and GAPDH levels (Table 2).

**Table 2:** Sequences of qPCR primers.

Gene	Forward	Reverse
*SMAD3*	GCCTTCTGGTGCTCCATCTC	AATAGCGCTGTCACTGAGGCA
*SMAD2*	TCATAGCTTGGATTTACAGCCAG	TTCTACCGTGGCATTTCGGTT
*ACTA2*	TATCCCCGGGACTAAGACGG	CACCATCACCCCCTGATGTC
*TAGLN*	AGTGGGGGAGGCTGACAT	TGGCAGGAAGGAGTGAAG
*TIMP1*	CCTTCTGCAATTCCGACCTC	GTATCCGCAGACACTCTCCA
*TIMP2*	CACCCAGAAGAAGAGCCTGA	TCTCTTGATGCAGGCGAAGA
*TIMP3*	GTCGCGTCTATGATGGCAAG	AAGCAAGGCAGGTAGTAGCA
*MMP1*	CTGGCCACAACTGCCAAATG	CTGTCCCTGAACAGCCCAGTACTTA
*MMP10*	ACTCTTTTGATGGCCCAGGA	GAGTGGCCAAGTTCATGAGC

### Western blotting

Protein samples were harvested at 4°C using 1X RIPA buffer containing fresh protease and phosphatase inhibitor cocktail (Thermo Fisher Scientific). Protein concentrations were determined using the Pierce BCA Protein Assay Kit. 50μg of each denatured HCASMC sample was loaded onto a 4–15% gradient SDS-PAGE gel (Bio-Rad). Samples were transferred to polyvinylidene difluoride membrane (Life Technologies) for 2h at 100V at 4°C and blocked with 5% milk in Tris-buffered saline and 0.05% TWEEN 20 (TBS-T, Sigma) for 1h at room temperature. Membranes were incubated with the following primary antibodies, mouse anti-GAPDH antibody (ab8245) was used as the loading control in all experiments. Anti-rabbit HRP (Sigma) or anti-mouse HRP (Sigma) secondary antibodies were used at a concentration of 1:10000 and diluted in 5% milk containing 0.05% Tween 20. Bands were detected using ECL western Blotting detection reagents (Pierce) per manufacturer’s instructions on the LI-COR Odyssey imaging system.

### RNA sequencing of SMAD3 regulated genes, network and pathway analyses

HCASMC (line 1508) were grown and *SMAD3* expression knocked down as above. Three experimental and three control samples were generated and sequenced on a HiSeq 4000 machine, 125 bp paired end reads. Reads were processed using rnaSeqFPro, a workflow for full processing of RNASeq data starting from fastq files. In brief, the quality control was performed using FastQC, mapping to the human genome hg19 was performed using STAR second pass mapping to increase the percentage of mapped reads, and counting was done with featureCounts using GENCODE gtf annotation. Next, rnaSeqFPro performed differential analysis using DESeq2, conducted principal component analysis and hierarchical clustering using standard R functions, plotPCA and heatmap.2 and generated graphs using gglot2. DESeq2 gave 493 differentially expressed (DE) genes (FDR ≤ 0.05).

The differentially expressed genelist was used to interrogate the Ingenuity Knowledge Base, identifying Canonical Pathways and Cardiovascular Disease, and Molecular and Cellular Functions category enrichments. There was also significant enrichment for Cardiovascular System Development and Function terms, and the genes attributed to the top subcategory “development of vasculature” were employed to build a *SMAD3* HCASMC network. Using only genes in this list, a network was created with connectivity supplied by the curated molecular interaction database of IPA. Visualization of this network was performed using Cytoscape open source software. Node color was mapped to log2 fold change with red representing genes that are downregulated along with *SMAD3* and green representing genes that are upregulated, and node size mapped to the number of interactions with other genes. Edges were colored to distinguish types forms of functional interactions.

### ChIP assay

Briefly, approximately 2e6 HCASMC cells were fixed with 1% formaldehyde and quenched by glycine. The cells were washed three times with PBS and then harvested in ChIP lysis buffer (50 mM Tris-HCl, pH 8, 5 mM EDTA, 0.5% SDS). Crosslinked chromatin was sheared for 3x1 min by sonication (Branson SFX250 Sonifier) before extensive centrifugation. Four volumes of ChIP dilution buffer (20 mM Tris-HCl, pH 8.0, 150 mM NaCl, 2 mM EDTA, 1% Triton X-100) was added to the supernatant. The resulted lysate was then incubated with Dynabeads Protein G (Thermo Scientific, 10009D) and antibodies at 4°C over-night. Beads were washed once with buffer 1 (20 mM Tris pH 8, 2 mM EDTA, 150 mM NaCl, 1% Triton X100, 0.1% SDS), once with buffer 2 (10 mM Tris pH 8, 1 mM EDTA, 500 mM NaCl, 1% Triton X100, 0.1% SDS), once with buffer 3 (10 mM Tris pH 8, 1 mM EDTA, 250 mM LiCl, 1% NP40, 1% sodium deoxycholate monohydrate) and twice with TE buffer. DNA was eluted by Chip elution buffer (0.1 M NaHCO3, 1% SDS, 20 μg/ml proteinase K). The elution was incubated at 65°C over-night and DNA was extracted with DNA purification kit (Zymo D4013). The purified DNA was assayed by quantitative PCR with ABI ViiA 7 and Power SYBR Green Master Mix (ABI 4368706) (Table 3).

**Table 3:** Sequences of ChIP PCR primers.

Gene	Forward primer	Reverse primer
*SERPINE1*	GACAGATCCAAGCAAGCCAG	GCCATTCTTATCTGCCCAGC
*COL1A1_SMAD3* locus	GCACAGACATACTTAGCGCC	CTTGACTGCTGGCTGGAATC
*COL1A1_TCF21* locus	CAGACATTCCCTCACCACCT	ATGATTCCAGTCCTGCTCCG
*ACTB*	TCTCCCCTCCTTTTGCGAAA	CAACGCCAAAACTCTCCCTC

### SMAD3 ChIPseq and follow-up analyses

ChIP was performed using the SMAD3 Abcam antibody ab28379 and HCASMC line 1508, and library was prepared using standard procedures. Briefly, DNA was prepared for end repair (Lucigen Endi-it, ER0720) and “A” tailing (NEB Klenow, M0212S), adaptor ligation (Promega, M180A), and library amplification (NEB, M0531S). ChIP-seq libraries were sequenced on HiSeq X10 for 150-bp paired-end sequencing. Quality control of ChIP-seq data was performed using *Fastqc*, and then low-quality bases and adaptor contamination were trimmed by *cutadapt*. After quality control and data filtering, data was mapped to hg19 using *BWA mem* algorithm. Duplicate reads were marked by *Picard Markduplicate* module and removed with unmapped reads by *samtools view -f 2 -F 1804*. *MACS2*.*1*.*1* was used for peaks calling with default parameters and input as control. We utilized the Genomic Regions Enrichment of Annotations Tool (GREAT 3.0) to analyze the detected peaks, with the parameter “Basal plus extension”, which is proximal: 5 kb upstream, 1 kb downstream, plus Distal: up to 1000 kb. Gene ontology from GREAT output were analyzed by DAVID. KEGG pathways, biological processes, molecular functions, and GAD disease enrichment analysis was carried out using default settings. The *HOMER findMotifsGenome*.*pl* script was employed to search for known TRANSFAC motifs and to generate de novo motifs. The *intersecBed* was used to find overlapped peaks between SMAD3 and TCF21. The filter used to cut off SMAD3 peaks is: fold change> 5 and -logq_value>10. Pooled TCF21 peaks were cut off with three different thresholds, liberal: fold change>5, -logq_value>25; standard: fold change>10, -logq_value>60; stringent: fold change>15, -logq_value>200. The fastq files of SMAD3 ChIPseq in A549 cell lines was extracted from SRR1014002 by *fastq-dump*. Similar methods were used in quality control, alignment, peak calling and intersection with SMAD3 peaks in HCASMC.

To test the relative binding of SMAD3 and TCF21 transcription factors in regions of the genome where their binding was colocalized by ChIPseq studies, we compared the two ChIPseq datasets by calculating overlapping regions using bedtools and creating distributions of normalized fold changes, i.e., relative read counts in peaks compared to background, with a relative scale 0–100 for overlapping or adjacent binding sites. The results were presented as a normalized fold change correlation plot indicating relative binding of the two transcription factors. This process was automated in an algorithm ChIPSeqCompare (https://github.com/milospjanic/ChIPSeqCompare), which investigates differential binding by two transcription factors that are hypothesized to interact via epigenetic modification or protein-protein-DNA interactions.

### ATAC-PCR assay

HCASMCs (passages 5–6) were cultured in normal media. Approximately 50,000 fresh cells were collected by centrifugation at 500g for 5min and washed with cold PBS. Nuclei-enriched fractions were extracted with cold lysis buffer (10 mM Tris–HCl, pH7.4, 10 mM NaCl, 3 mM MgCl2 and 0.1% IGEPAL) and the pellets were resuspended in transposition reaction buffer (20 mM Tris-Cl pH7.5, 10 mM MgCl2, 20% Dimethylformamide) and Tn5 transposase (Illumina Nextera). Transposition reactions were incubated at 37°C for 30 min, followed by DNA purification using the DNA Clean-up and Concentration kit (Zymo D4013). The genomic DNA was extracted using Quick-DNA Microprep Kit (Zymo D3020). The purified DNA was quantified by qPCR with ABI ViiA 7 and Power SYBR Green Master Mix (ABI 4368706) and normalized by genomic DNA.

### Assessment of SMAD3 causality and direction of effect for CAD

To test directionality of effect of *SMAD3 cis*-risk alleles in HCASMC on *SMAD3* gene expression, we investigated *SMAD3* eQTL data emanating from a genome-wide association of gene expression with imputed common variation identified in 52 HCASMC studied with whole genome RNA sequencing and 30x whole genome sequencing [59]. Significance of association of variance in *SMAD3* expression with genotype was evaluated by regression analysis, the results were visualized with HCASMCeQTLviewer (https://github.com/milospjanic/HCASMCeQTLviewer), a combined bash/R/awk script that outputs the directionality of effect for any SNP-gene association in HCASMC.

To test causality and directionality of the *SMAD3* gene in CAD in the context of multiple cis-acting eQTLs, we investigated the directionality of change of expression level with a number of risk GWAS SNPs. This analysis employed CAD GWAS data from a recent meta-analysis [7] to select risk SNPs and define risk alleles and HCASMC eQTL data to perform regression analysis. To facilitate this analysis, we developed a custom algorithm to analyze whole genome sequencing and expression data in HCASMC. We developed GeneCausalityTest for coronary artery disease (https://github.com/milospjanic/GeneCausalityTestCAD), a combined bash/awk/R script for defining causality of a gene for a given trait, in this case CAD, given the directionality of change of expression level with the increasing number of risk GWAS SNPs. This correlation was improved by resolving local haplotype structure, creating a sample correlation matrix and averaging on samples with unique local haplotype profile. This approach improved correlations made by GeneCausalityTest for defining causality of a gene for CAD, especially in the local regions of strong linkage disequilibrium. This analysis provided output for the directionality of expression change with the increasing number of risk SNPs and used CAD GWAS data (Nelson et al.) and HCASMC eQTL data for regression analysis. For correlating the effect sizes of *SMAD3* variants and *SMAD3* gene expression, the CAD GWAS summary data from Nelson et al. and *cis*-eQTL summary data from STARNET were obtained from www.cardiogramplusc4d.org and dbGaP (phs001203.v1.p1), respectively [7, 60]. The files were loaded and processed in R using subset and merge functions to obtain an overlapping variant list at GWAS *P*<1E-04 and nominal *cis*-eQTL *P*<0.05. The beta coefficients (negative log odds ratio (OR)) of these variants were plotted using ggplot and a linear mixed model was used to compute a smooth local regression. Pearson correlation coefficient *r and p*-value of significance were calculated using cor.test in R. This method has been standardized in an algorithm named UniqueHaplotypeTestCAD (https://github.com/milospjanic/UniqueHaplotypeTestCAD).

### Dual Luciferase reporter assays

A dual Luciferase assay (Promega, #E1910) was used to measure the luciferase and renilla activity in the transfected cells. Five hours after transfection, growth media was removed, and cells were washed with 1X PBS. For cell lysis, cells were incubated with 100 μl of 1X Passive cell lysis buffer for 20 minutes at room temperature. 10 μl of the cell lysate was transferred to 96 well white flat bottom assay plates (Costar, #3912). Luciferase assays were performed with SpectramaxL Microplate luminometer with SoftMax Pro software. First, 100 μl of Luciferase assay substrate was injected into the lysate and luciferase activity was measured for 10 sec. Subsequently, 100 μl of Stop and Glo reagent was injected to the lysate to stop the luciferase activity and catalyze the renilla reaction. Renilla activity was measured for 5 seconds following 1.6 second incubation. HCASMCs and A7R5s were transfected with either SMAD binding element-luciferase reporter plasmid (pSBE4 luc), pGL3-hSM22-325-luc plasmid, pGL3-luc or the pBV-luc constructs and stimulated with *SMAD3* (pRK5F *SMAD3*) and *TCF21* (pCMV6 XL4) cDNAs. The Renilla luciferase reporter plasmid was used as the internal control of transfection efficiency.

### Statistical analyses

Experiments were performed by the investigators blinded to the treatments/conditions during the data collection and analysis, using at least two independent biological replicates and treatments/conditions in technical triplicate. For the statistical analyses not discussed above, methods were as follows. R or GraphPad Prism 7.0 was used for statistical analysis. For motif and gene enrichment analyses, we used the *cumulative binomial distribution* test. For overlapping of genomic regions or gene sets, we used *Fisher’s exac*t test and/or the *hypergeometric* test, as indicated. For comparisons between two groups of equal variance, an unpaired two-tailed Student’s *t-test* was performed or in cases of unequal variance a Welch’s unequal variances t-test was performed, as indicated. *P* values <0.05 were considered statistically significant. For multiple comparison testing, one-way analysis of variance (ANOVA) accompanied by Tukey’s *post hoc* test were used as appropriate. All error bars represent standard error of the mean (SE).

## Supporting information

S1 Table IPA analysis of genes identified in siTCF21 knockdown and RNA-Seq studies of differentially expressed genes shows enrichment in Cardiovascular Disease phenotype genes. (XLSX)

S2 Table Comparison of siSMAD3 RNAseq top processes in IPA (Physiologic System Dvlp and Fxn), compared to recent CAD GWAS IPA analysis of predicted CAD GWAS causal genes. (XLSX)

S3 Table IPA analysis of genes identified in siTCF21 knockdown-RNA-Seq studies, identified CV System Development and Function terms. (XLSX)

S4 Table IPA analysis of DE genes identified in siTCF21 knockdown—RNA-Seq studies, molecular and cellular function pathways. (XLSX)

S5 Table IPA analysis of DE genes identified in siSMAD3 knockdown—RNA-Seq studies identified a Vascular Development regulatory network. (XLSX)

S1 Fig *SMAD3* promotes expression of differentiation markers, migration, and inhibits proliferation of HCASMC. A) HCASMC were treated with *SMAD3*-specific (S3 KD) or scrambled sequence (SCR) siRNA, and evaluated by western blot analysis for expression of lineage markers *ACTA2* and *TAGLN* and control protein GAPDH. Identical cultures of HCASMC were transfected with a *SMAD3* encoding expression plasmid (SMAD3) or control plasmid (CTRL), and cells similarly evaluated by western analysis for SMC marker and GAPDH protein levels. B, C) Expression of differentiation markers *ACTA2* and *TAGLN* was also evaluated by quantitative immunofluorescence in HCASMC with knockdown (S3 KD) or over expression (SMAD3) of SMAD3. D) Migration of HCASMC was evaluated with a gap closure assay, and E) proliferation was evaluated with a EdU assay, employing the same knockdown and over-expression models as described.
(TIF)

S2 Fig SMAD3 has an opposing effect on expression of many of the genes in the previously described TCF21 “vascular disease” transcriptional network. A) RNAseq data of HCASMC studied under control and si*TCF21* knockdown conditions was analyzed with DEseq to identify differentially regulated genes. Analysis of these identified genes with the Ingenuity analysis software identified interactions of a number of genes with identified roles in vascular disease [34]. These genes were employed to generate a TCF21 transcriptional network, as visualized with Cytoscape. Node color was mapped to log fold change with green representing genes that are downregulated along with TCF21 and red representing genes that are upregulated, node size was mapped to absolute expression value in control cells, and font size to enrichment Q-value. Edges are colored to distinguish types of interactions. Green edges represent functional interaction (protein-protein binding, protein modification, molecular cleavage, phosphorylation, and protein-DNA interactions); magenta edges represent gene expression (expression and transcription) relationships; red edges represent activation; and blue edges inhibition. B) Changes in gene expression resulting from siRNA knockdown of *SMAD3* in HCASMC were mapped onto the TCF21 network by changing the node color to reflect changes in gene expression using the same color scheme as employed for TCF21.
(TIF)

S3 Fig Regulation of matrix protein gene expression by SMAD3. **A)** Expression levels of *TIMP1*, *TIMP2*, *TIMP3*, *MMP1*, and *MMP10* were measured in HCASMC transfected with either specific siRNA (S3 KD) or scrambled RNA (SCR), and expression levels measured with qRT-PCR. B) Similar experiments were performed with *SMAD3* over-expression (*SMAD3*) and control transfections (CTRL), and gene expression measured.
(TIF)

S4 Fig SMAD3 ChIPseq analyses. A) ChIP-PCR confirmation of SMAD3 binding sites at *TAGLN*, *CNN1*, *COL1A1*, and *SERPINE1* loci identified by ChIPseq studies. B) DAVID Gene Ontology molecular function analysis of all SMAD3 target genes identified by GREAT with basal plus extension mode. C) GO analysis of target genes (GREAT output) of all SMAD3 peaks that colocalize with TCF21 peaks (as described for Fig 4E).
(TIF)
