# Supplementary figures and images for: Coronary artery disease genes SMAD3 and TCF21 promote opposing interactive genetic programs that regulate smooth muscle cell differentiation and disease risk
Source: PLoS Genet. 2018 Oct 11;14(10):e1007681. doi: 10.1371/journal.pgen.1007681 (PMC6198989; doi:10.1371/journal.pgen.1007681)

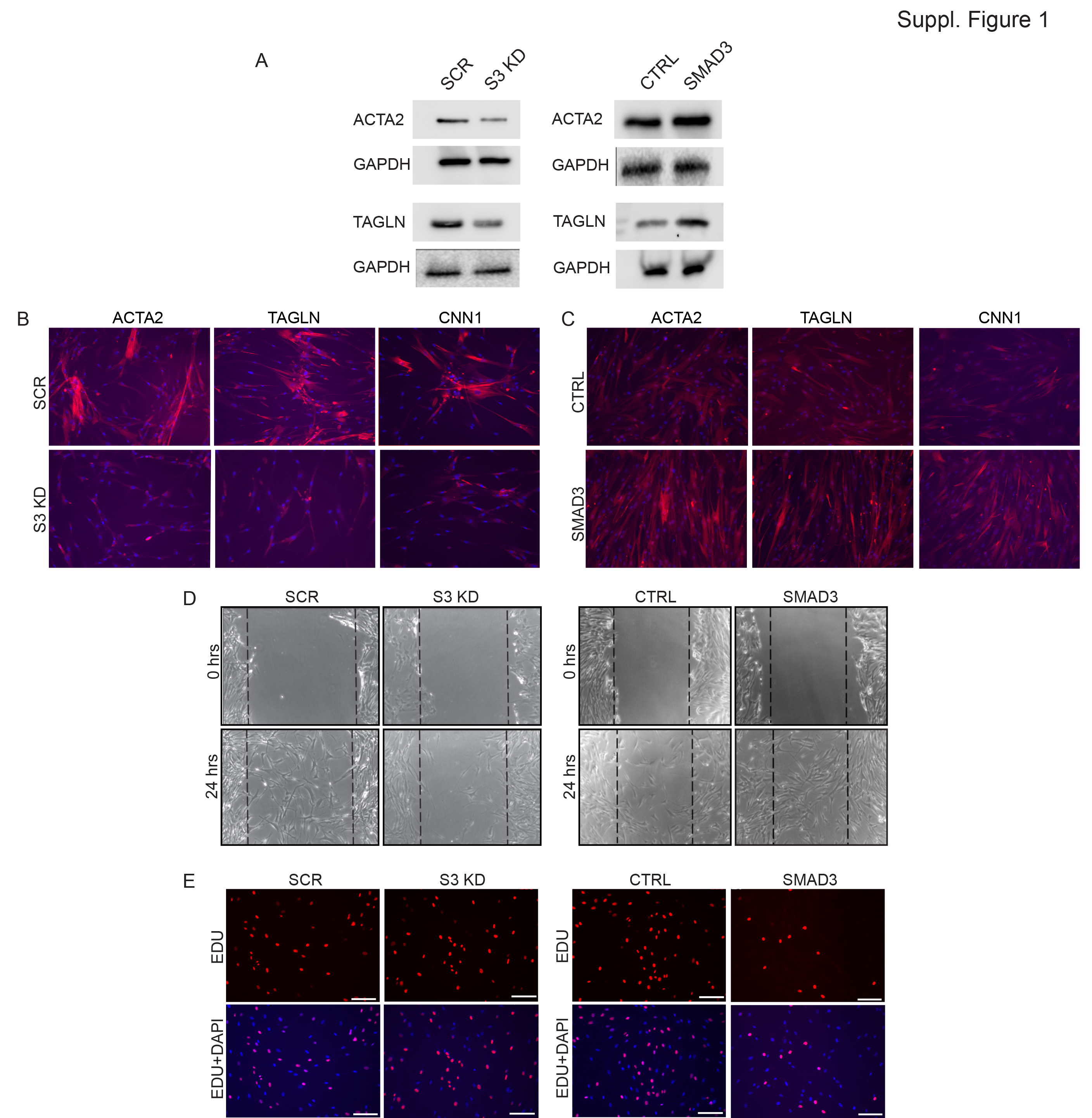

Supplement: S1 Fig — A) HCASMC were treated with SMAD3-specific (S3 KD) or scrambled sequence (SCR) siRNA, and evaluated by western blot analysis for expression of lineage markers ACTA2 and TAGLN and control protein GAPDH. Identical cultures of HCASMC were transfected with a SMAD3 encoding expression plasmid (SMAD3) or control plasmid (CTRL), and cells similarly evaluated by western analysis for SMC marker and GAPDH protein levels. B, C) Expression of differentiation markers ACTA2 and TAGLN was also evaluated by quantitative immunofluorescence in HCASMC with knockdown (S3 KD) or over expression (SMAD3) of SMAD3. D) Migration of HCASMC was evaluated with a gap closure assay, and E) proliferation was evaluated with a EdU assay, employing the same knockdown and over-expression models as described. (TIF) [file pgen.1007681.s006.tif]

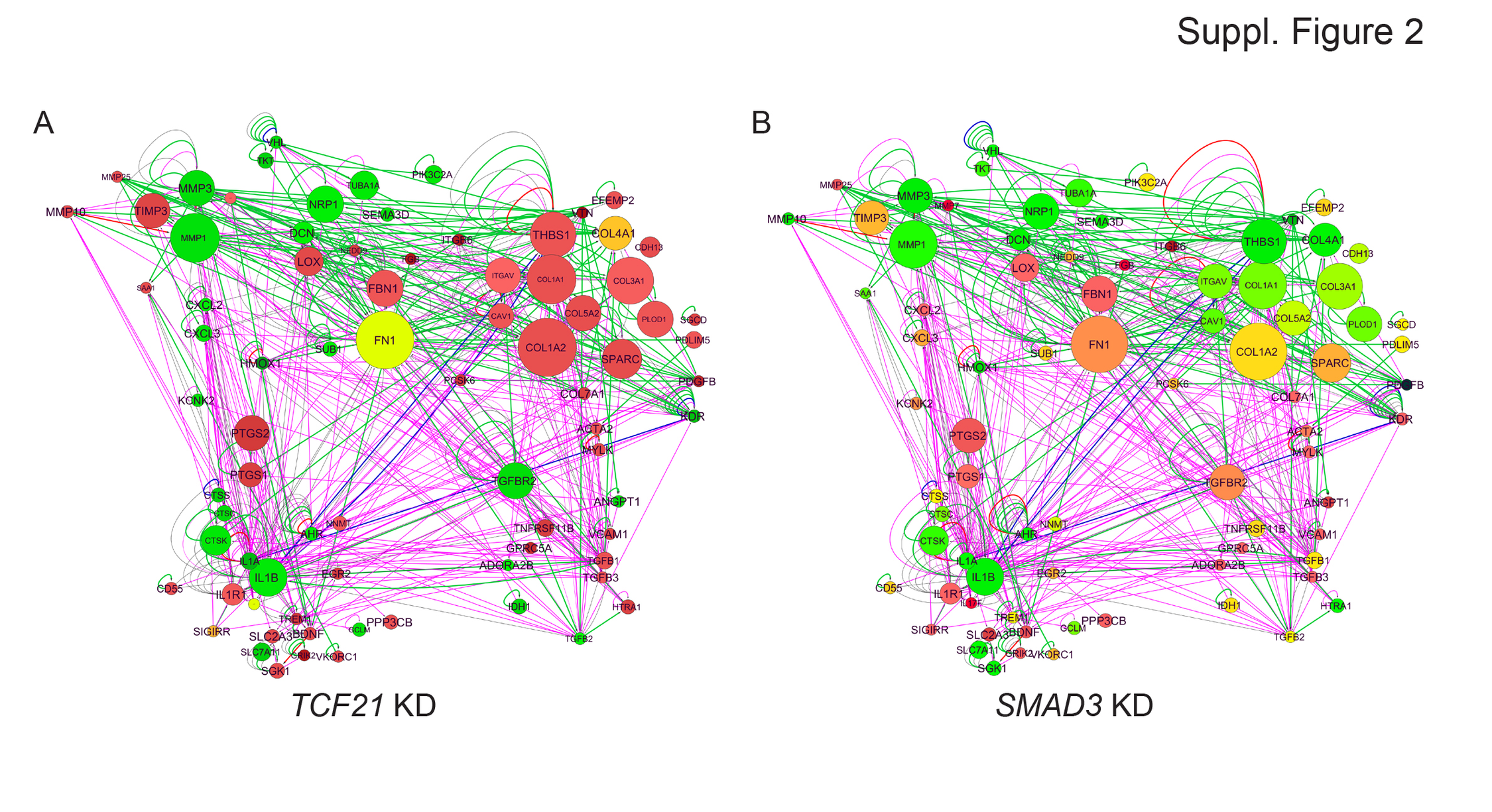

Supplement: S2 Fig — A) RNAseq data of HCASMC studied under control and siTCF21 knockdown conditions was analyzed with DEseq to identify differentially regulated genes. Analysis of these identified genes with the Ingenuity analysis software identified interactions of a number of genes with identified roles in vascular disease [34]. These genes were employed to generate a TCF21 transcriptional network, as visualized with Cytoscape. Node color was mapped to log fold change with green representing genes that are downregulated along with TCF21 and red representing genes that are upregulated, node size was mapped to absolute expression value in control cells, and font size to enrichment Q-value. Edges are colored to distinguish types of interactions. Green edges represent functional interaction (protein-protein binding, protein modification, molecular cleavage, phosphorylation, and protein-DNA interactions); magenta edges represent gene expression (expression and transcription) relationships; red edges represent activation; and blue edges inhibition. B) Changes in gene expression resulting from siRNA knockdown of SMAD3 in HCASMC were mapped onto the TCF21 network by changing the node color to reflect changes in gene expression using the same color scheme as employed for TCF21. (TIF) [file pgen.1007681.s007.tif]

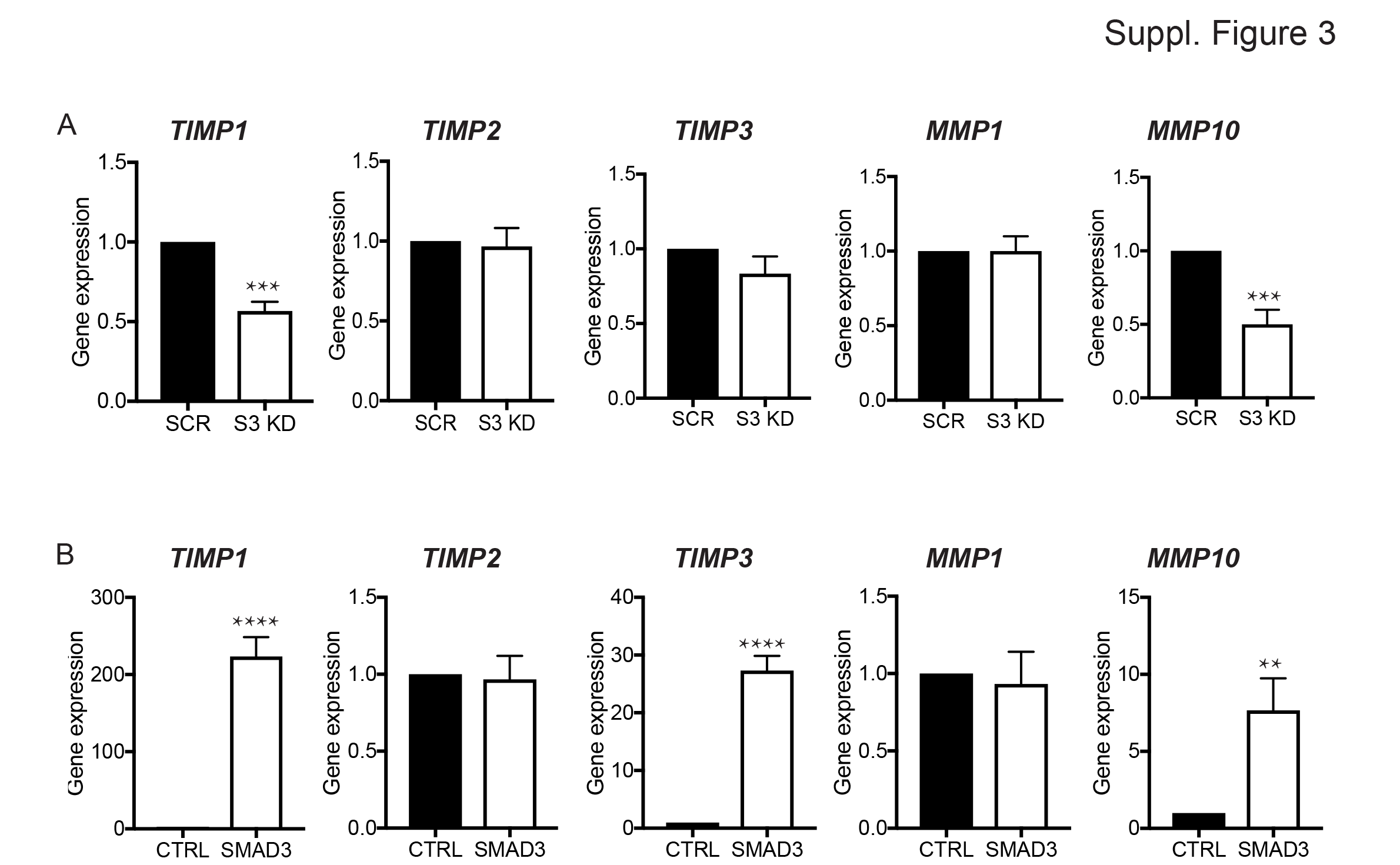

Supplement: S3 Fig — A) Expression levels of TIMP1, TIMP2, TIMP3, MMP1, and MMP10 were measured in HCASMC transfected with either specific siRNA (S3 KD) or scrambled RNA (SCR), and expression levels measured with qRT-PCR. B) Similar experiments were performed with SMAD3 over-expression (SMAD3) and control transfections (CTRL), and gene expression measured. (TIF) [file pgen.1007681.s008.tif]

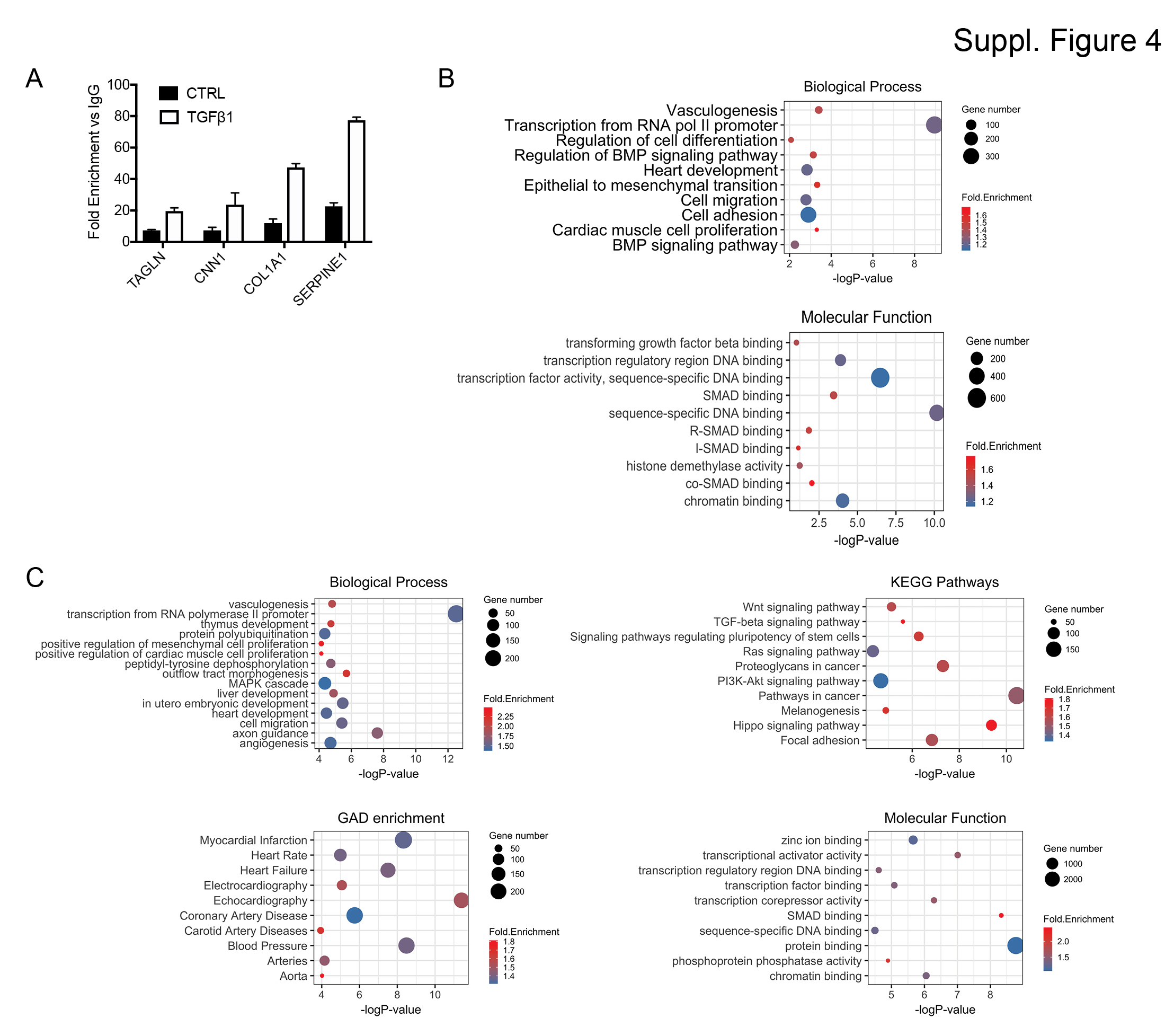

Supplement: S4 Fig — A) ChIP-PCR confirmation of SMAD3 binding sites at TAGLN, CNN1, COL1A1, and SERPINE1 loci identified by ChIPseq studies. B) DAVID Gene Ontology molecular function analysis of all SMAD3 target genes identified by GREAT with basal plus extension mode. C) GO analysis of target genes (GREAT output) of all SMAD3 peaks that colocalize with TCF21 peaks (as described for Fig 4E). (TIF) [file pgen.1007681.s009.tif]
